# Late shellmound occupation in southern Brazil: A multi-proxy study of the Galheta IV archaeological site

**DOI:** 10.1371/journal.pone.0300684

**Published:** 2024-03-21

**Authors:** Jessica Mendes Cardoso, Fabiana Merencio, Ximena Villagran, Veronica Wesolowski, Renata Estevam, Benjamin T. Fuller, Paulo DeBlasis, Simon Pierre-Gilson, Danaé Guiserix, Pauline Méjean, Levy Figuti, Deisi Farias, Geovan Guimaraes, Andre Strauss, Klervia Jaouen

**Affiliations:** 1 Géosciences Environnement Toulouse, Observatoire Midi Pyrénées, UMR 5563, CNRS, Toulouse, France; 2 Museu de Arqueologia e Etnologia, Universidade de São Paulo, São Paulo, Brazil; 3 Universidade Federal de Santa Catarina, Florianópolis, Brazil; 4 Universidad Las Palmas de Gran Canaria, Las Palmas de Gran Canaria, Spain; 5 École Normale Supérieure de Lyon, Lyon, France; 6 Grupo de Pesquisa em Educação Patrimonial e Arqueologia, Universidade do Sul de Santa Catarina, Tubarão, Brazil; New York State Museum, UNITED STATES

## Abstract

Brazilian coastal archaeology is renowned for its numerous and large shellmounds *(sambaquis*), which had been continuously occupied from at least 8000 to 1000 years cal BP. However, changes in their structure and material culture in the late Holocene have led to different hypotheses concerning their ecological and cultural changes. The archaeological site Galheta IV (ca. 1300 to 500 years cal BP) offers new insights into the complexity of the late coastal occupation in southern Brazil. Our attempt was to determine whether Galheta IV can be classified as a *sambaqui* site, or if it belongs to a Southern *proto*-*Jê* settlement. Here, we reassessed Galheta’s collections and applied a multi-proxy approach using: new ^14^C dates, zooarchaeology, δ^13^C and δ^15^N isotopes in bulk collagen and ^87^Sr/^86^Sr_enamel_ isotopic ratios from eight human individuals, ceramics analysis, and FTIR. The results indicate an intense exploitation of marine resources, with an area designated for processing animals located at the opposite side of the funerary areas. Bone tools and specific species of animals were found as burial accompaniments. No evidence of human cremations was detected. ^87^Sr/^86^Sr results indicate that the eight human individuals always lived on the coast, and did not come from the inland. The pottery analysis confirms the association with Itararé-Taquara, but contrary to what was assumed by previous studies, the pottery seems related to other coastal sites, and not to the highlands. In light of these findings, we propose that Galheta IV can be considered a funerary mound resulting from long and continuous interactions between shellmound and Southern *proto*-*Jê* populations. This study not only enhances our understanding of the late coastal occupation dynamics in southern Brazil but also underscores its importance in reshaping current interpretations of shellmound cultural changes over time.

## Introduction

Shellmounds and shellmiddens are among the oldest and most widespread features of coastal occupation by modern humans. These structures, whether intentionally built or fortuitously accumulated, provide insights into the lifestyles, adaptations, and occupations of the associated populations in various environmental and cultural contexts [1–4]. In South America, specifically in southern Brazil, human groups occupied the region close to the Atlantic Ocean from at least 8000 to 1000 years cal BP [5–9]. Over this period, thousands of shellmounds were constructed, some of which are over 30 meters in height and 400–500 meters in length and width [10].

The southern Brazilian shellmounds, locally known as *sambaquis*, are the evidence of a long-term occupation, with layers of shells, bones of vertebrate animals (primarily fishes), plant remains (mainly micro-remains), hearths, combustion debris, and artifacts made with lithic, bones and shells. The *sambaquis* were exceptional territorial markers with multiple functions, but the largest and most studied sites often served as cemeteries, formed from several mounds overlapping successive funerary areas [5, 11, 12]. The location of shellmounds near aquatic ecosystems and the intentional habit of piling up shells often suggest that human groups belonged to the same culture. However, variations have been observed in the same regions, and also between different areas, leading to new assumptions about the complexity of *sambaquis*, particularly in the late periods of occupation [13–17].

In Santa Catarina State, there was a noticeable decrease in shellmound activity starting from 2200–2100 years cal BP, followed by a discontinuity process in their construction [12, 18]. Dark sediment layers are visible on top of the shellmounds; new, smaller sites formed by faunal bones and, sometimes, fragments of ceramics appeared. Two possible explanations were advanced to account for these changes. The first hypothesis is that this phenomenon resulted from the gradual expansion of new ceramist groups associated with the Southern *proto-Jê* populations, who migrated from inland regions to the coastal areas over an extended period, eventually encountering the *sambaquis* populations [13]. The second explanation suggests that the progressive abandonment of the traditional *sambaqui*-building practices was influenced by environmental changes, potentially triggered by climate fluctuations or sea level variations [6, 19–24]. Alternatively, it is plausible that a combination of environmental and cultural factors contributed to the decrease of these sites in the region [18, 25].

This study aims to contribute to this ongoing debate by re-evaluating and analyzing the archaeological collections from Galheta IV (ca. 1300 to 500 years cal BP [13]), located in the municipality of Laguna, in Southern Santa Catarina State. Galheta IV is a funerary site that exhibits both continuity with the shellmound occupation, particularly with regard to the settlement in the landscape and lithic technology, and discontinuity, including the stratigraphic composition and the presence of ceramic fragments, presumably produced by the ancestrals of *Jê*-speaking groups that inhabit the area today. In particular, we attempt to determine whether Galheta IV can be classified as a *sambaqui* site, or if it belongs to the archaeological Southern *proto*-*Jê* associated culture, by considering several lines of evidence, including: chronology, zooarchaeology, diet, geographical provenance, funerary practices, and pottery. To achieve this, we employed a multi-proxy approach, which included zooarchaeological analysis, isotopic measurements, Fourier-Transform Infrared Spectroscopy (FTIR), and analysis of the ceramics found on the site. We also conducted direct radiocarbon dating on five human individuals to refine the chronology of occupation and investigate the potential arrival of new populations to the coastal region.

Stable isotope ratios of carbon and nitrogen allow us to quantify the different food categories in the diet: terrestrial *versus* marine resources, and plant *versus* animal product consumption [26, 27]. The discussion about diet at Galheta IV started by considering the δ^13^C and δ^15^N analysis from bones of seven individuals from the site [28], but the absence of analyses on the associated fauna prevented further interpretations. By incorporating zooarchaeological data into the analysis of a complex archaeological site, such as Galheta IV, we are able to better understand the environmental context, to quantify the animal remains that could have been consumed, track their distribution within the site, and determine whether bones and teeth were used as tools.

Archaeogenetic analysis of one individual buried at Galheta IV showed greater affinity with *sambaquis* populations rather than with *Jê* groups [25]. However, there is no information available about the geographical provenance of the other individuals buried at the site. To do so, we applied ^87^Sr/^86^Sr isotopic ratios of bioapatite of both humans and animals, taking into account the geological context and potential bias caused by seafood consumption on isotope analyses. The goal was to identify potential Southern *proto*-*Jê* migrants coming from the hinterland. Strontium isotope ratios of foods and animal tissues are dependent on the local geology [29]. Plants and seafood are the primary sources of strontium in human diets [30]. Strontium isotope ratios are uniform in the ocean (^87^Sr/^86^Sr = 0.7092). Seafood consumption can therefore bias mobility reconstruction. It is thus crucial to evaluate the reliance on marine resources of humans when searching for their geographical origin [29, 31].

Information about the funerary practices has been documented [13], raising the hypothesis that burials in Galheta IV could have been burned, representing cremations like the burials registered in Southern *proto*-*Jê* archaeological sites [32, 33]. The ceramics found at the site also suggest a relationship with the Itararé-Taquara Tradition [13]. Our study provided the opportunity to perform a detailed analysis of burned human and fauna bones, as well as a systematic and comparative analysis of pottery fragments.

In summary, we employed a multi-proxy approach to achieve a better understanding of Galheta IV and the intricate social and environmental context of the late Holocene in southern Brazil.

### Study area and late shellmounds occupation

**Fig 1:**
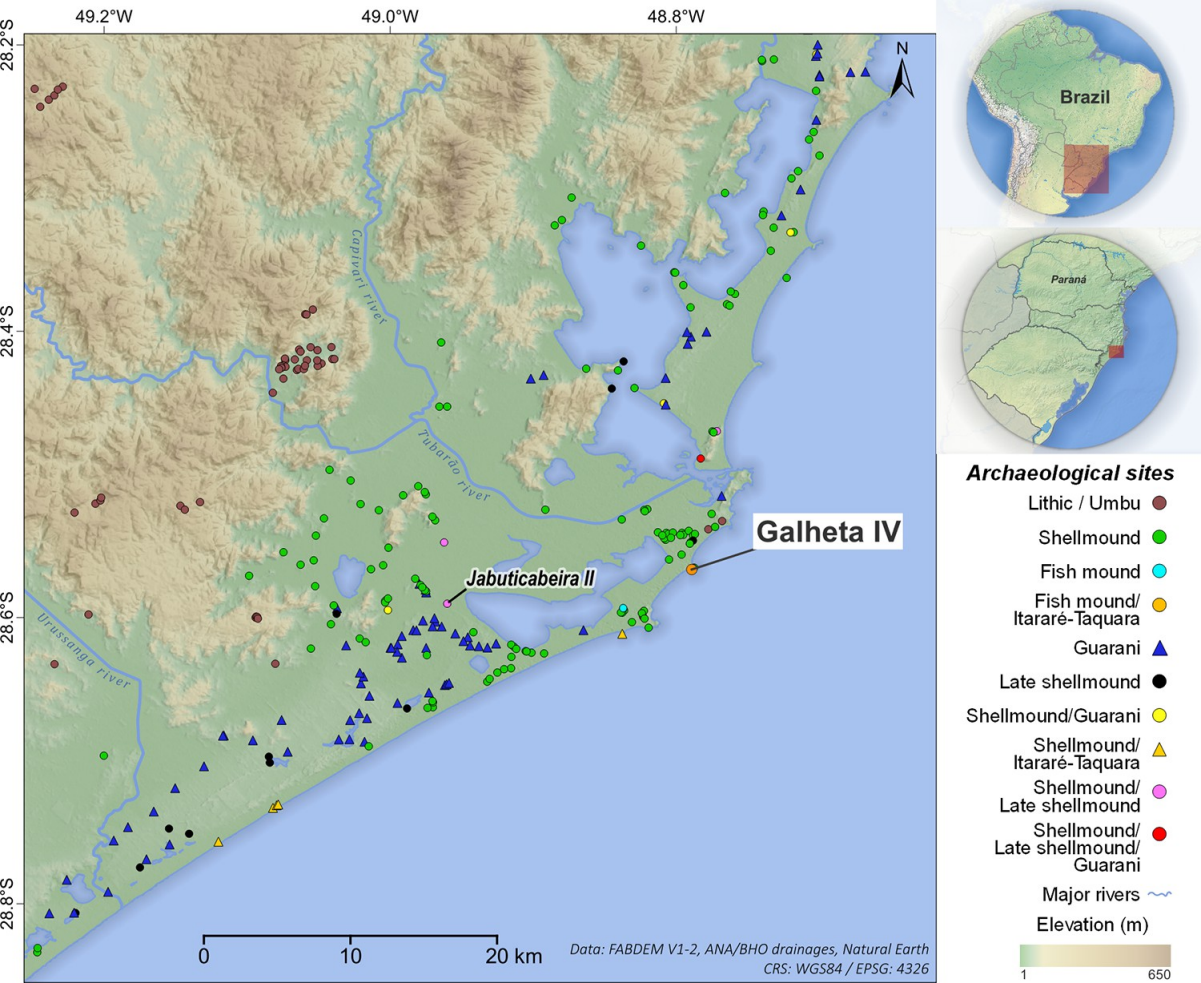
Location of Galheta IV archaeological site and other settlements of Southern Santa Catarina. Made with free vector and raster map data. Background: Natural Earth [34]; Digital elevation model: FABDEM V1-2 [35]; Rivers: Brazilian National Water Agency (ANA) [36]. All utilized geographical data are under the Creative Commons Attribution License (CC BY 4.0). Software: QGIS 3.28. Note: Own elaboration.

This research focuses on the southern coast of Brazil, in the state of Santa Catarina (Fig 1), an area characterized by the well-known and monumental coastal shellmounds that have been studied since the 19^th^ century [6, 11]. While there has been significant progress in understanding the populations associated with the larger shellmounds, smaller sites from the final period of occupation have received less attention. There are still many unanswered questions regarding their interactions with different cultures, the introduction/adoption of ceramics, food production in coastal areas, and the disappearance of these populations.

The oldest *sambaquis* in this region date back to approximately 7500 years cal BP, with a significant abundance of active shellmounds recorded around 3000 years cal BP. However, around 2200 years cal BP, the composition of these sites changed [12, 18, 37, 38]. At the same time, the continuity of some elements was noticed, such as mound architecture, insertion in the landscape, exploitation of aquatic resources and lithic technology [14, 39].

The changes observed in the later stages of shellmound occupations are related to the replacement of shell layers interspersed with thin lenses of organic material by thick, dark sandy layers with fewer or no shells. This is also observed in a variety of sites that emerged in the region after ca. 2400 years cal BP: 1) **late *sambaquis*** (ca. 2400–900 years cal BP), which are organic-rich shallow shell sites with low visibility and no associated ceramic; 2) **fish mounds/“mixed” sites** (ca. 1300–500 years cal BP), characterized by thick dark layers, formed by fish bones and charcoal, and the presence of Itararé-Taquara ceramics; and lastly, 3) **shellmounds with Itararé-Taquara ceramic** (ca. 1000–500 years cal BP), that differ from fish mounds by the fact that they present a stratigraphy marked by thin layers of shells with dark sediment, rich in organic matter [14, 39].

There are multiple factors that could have contributed to the changes observed in the Late Holocene archaeological record. From an environmental perspective, the sea-level regression [19–21] and/or the local sedimentary processes [22] caused a reduction in the connection between lagoons and the ocean, altering the salinity of estuaries and gradually increasing the silting up of lagoons and wetlands. This condition may have reduced the availability of mollusks such as *Anomalocardia flexuosa* [23], which were a major resource used to construct local *sambaquis*. At the same time, strong winds and an increase in unconsolidated sands may have influenced the decrease of active shellmounds located close to the sand barrier and paleolagoon [24].

The presence of pottery in certain late coastal sites has sparked a discussion regarding the interaction between *sambaqui* culture and other groups, particularly the Southern *proto*-*Jê*. The Itararé-Taquara pottery tradition is associated with pre-colonial groups linked to Southern *proto*-*Jê* populations [40, 41], and those populations are attributed to the expansion of *Jê*-speaking groups from Central Brazil, historically and ethnographically known as Kaingang and Laklãnõ-Xokleng in the south [42, 43]. The Itararé-Taquara ceramics occur over a wide area, from southern Brazil and parts of the central-western and southeastern Brazil to northeastern Argentina. This vast area includes distinct ecotones: beaches, river valleys, hillsides, and mountaintops [44–47]. This pottery tradition was found at the Santa Catarina coastal plain during the final Holocene, likely originating from the southern plateau, hillside, and surrounding areas [13].

The Southern *proto*-*Jê* people had a wide cultural diversity, evidenced by their material culture and variety of settlements. They built earth-engineered structures such as underground houses and circular structures, and also occupied open-air sites and rock shelters with burials and/or rock inscriptions. They produced ceramics, practiced cremation of the dead, and their economy involved hunting, fishing, gathering, and food production, with emphasis on the consumption of *pinhão*, the seed of *Araucaria angustifolia* [32, 44, 48–51].

Significant environmental changes also took place on the plateau of Santa Catarina during the Holocene, particularly around 1500 years cal BP. It was during this time that the Araucaria Forest, also known as *Mata Ombrofila de Altitude* or *Mata de Araucárias*, began to expand, gradually extending into the open fields. This expansion was accompanied by a shift towards a warmer and more humid climate, following a previous colder and drier phase [52]. Some studies [53, 54] found genetic, paleoecological, and palynological evidence that demonstrate the influence of human groups speaking the *Jê* language on the dispersion of the Araucaria Forest. The forest’s expansion exceeds its natural geographic limits, spreading widely in the region, particularly in areas more densely occupied by Southern *Jê* populations.

Archaeogenetic analyses of Amerindian populations [25, 55] indicate that human skeletal remains of the *sambaqui* Jabuticabeira II, located only 20 km from Galheta IV, have a genetic affinity with the present-day Kaingang, who are descendants of the Southern *proto*-*Jê*. This genetic data corroborates the idea that these shellmounds societies share a common *Jê* ancestry and had interbred with those populations in recent times [25]. Our study aims to build upon this genetic evidence by exploring archaeological traces of *Jê* interaction with the *sambaquis* at Galheta, seeking to understand the identities of the occupants during this historical period.

## Materials and methods

The archaeological site of Galheta IV is situated on the Ponta da Galheta, a granite rocky shore covered with aeolian sand dunes, facing the Atlantic Ocean. It is one of three shellmounds on this rocky shore, with Galheta I and Galheta II being the other two. Previous research [39] has identified Galheta IV as a fishmound or mixed site, based on its stratigraphy, composition, and formation processes. Excavated in the early 2000’s, Galheta IV is a unique site, characterized by its funerary function, with eight human burials found, as well as a significant amount of vertebrate faunal bones and lithic industry. Its stratigraphic features distinguish it from the older shellmounds, as it lacks shell layers and contains ceramic fragments associated with the Southern *proto-Jê*.

**Fig 2:**
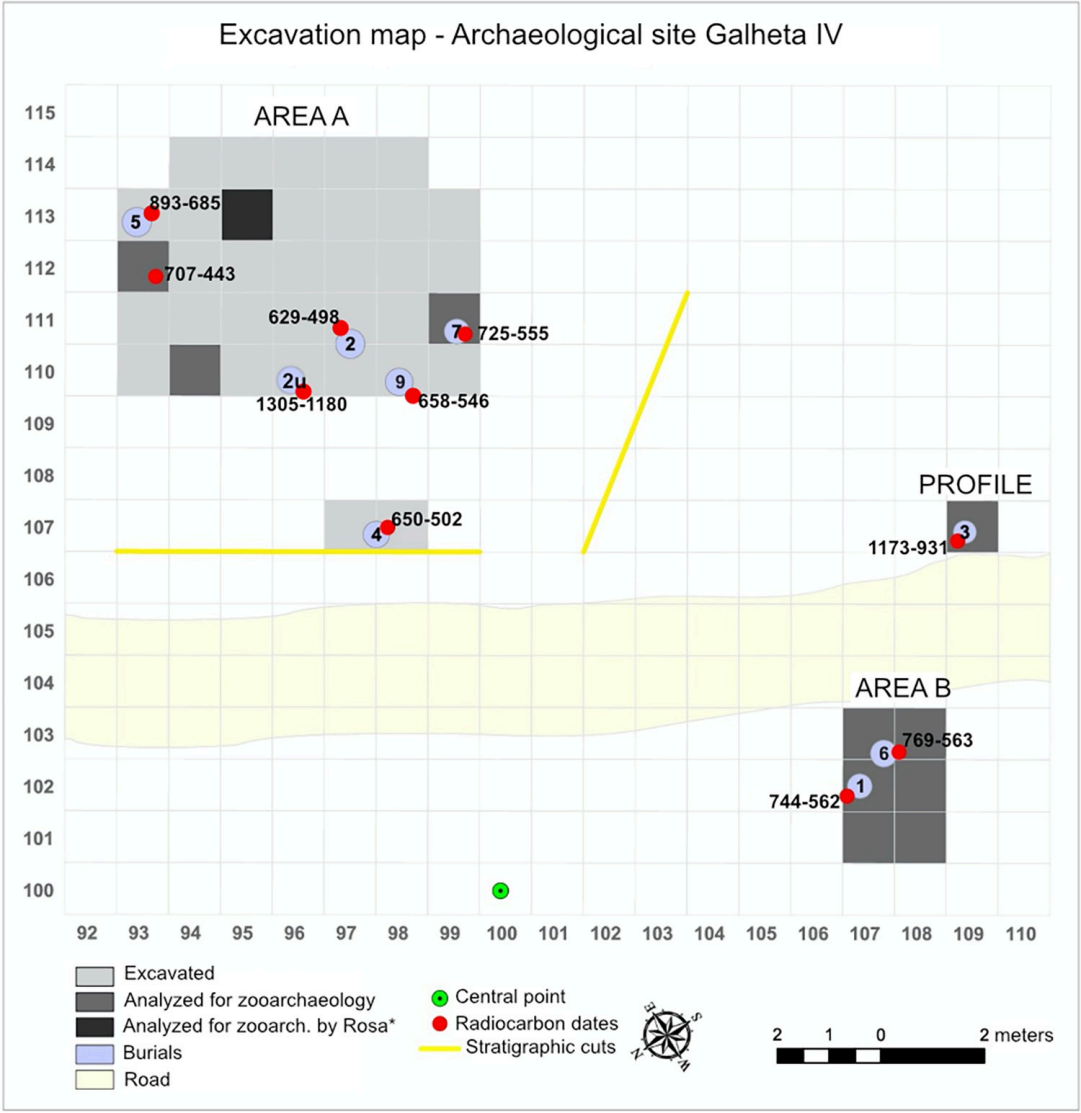
Excavation grid map of Galheta IV during the “*Sambaquis e Paisagem*” project. Including the selected units for zooarchaeological analyses, location of burials and calibrated radiocarbon dates. The burial 2u is the Burial unknown 2, tooth isolated found in fauna samples. Note: Own elaboration.

For this research, we conducted a comprehensive study of the collections excavated at the Galheta IV site. Our investigation involved consulting primary field documentation and reconstructing the excavation grid map for all field campaigns (see Fig 2). Excavations were concentrated in three distinct areas: ’Area A,’ ’Area B,’ and ’Profile.’ These areas were excavated in 1 m^2^ square units, with artificial levels of 10 cm, collectively covering an area of 42 m^2^.

The site yielded nine human burials, of which eight were excavated. Estimated ages at death ranged from approximately 14 to over 25 years old. Determination of biological sex and precise age proved challenging due to poor preservation and/or the absence of diagnostic skeletal elements. The biological sex of two individuals (Burial 3b and 5) were identified as female through DNA analysis [25]. Additionally, human bone and teeth were retrieved from zooarchaeological samples. Two detached human teeth, lacking funerary context (designated as Burial unknown 1 and Burial unknown 2), were also included in our research (Table 1). Detailed information about human burials can be consulted at the S2 File.

**Table 1:** List of human burials and type of analysis performed in this study and the literature.

Sample	Body deposition	Age	Sex	^14^C	δ^13^C/ δ^15^N_bone_	δ^13^C/ δ^15^N_dentine_	^87^Sr/^86^Sr_enamel_	FTIR
**Burial 1**	primary/ simple	17 to 19 y	undetermined	yes	yes	yes	yes	yes
**Burial 2**	secondary/simple	Undetermined	undetermined	yes	yes	no	no	yes
**Burial 3**	secondary/multiple of probably 4	3b: 14 to 16 y	3b: female	yes	no (burned bones)	no (burned bones)	yes (3b)	yes (3b)
**Burial 4**	primary/ simple	± 20 y	undetermined	yes	yes	yes	yes	yes
**Burial 5**	unclear	± 21 y	female	yes	yes	yes	yes	yes
**Burial 6**	primary/ simple	>25 y	undetermined	yes	yes	no	no	yes
**Burial 7**	unclear	19 to 20 y	undetermined	yes	yes	yes	yes	yes
**Burial 8**	not excavated							
**Burial 9**	unclear	undetermined	undetermined	yes	yes	no	no	yes
**Burial unknown 1**	no funerary context	undetermined	undetermined	no	no	yes	yes	no
**Burial unknown 2**	no funerary context	undetermined	undetermined	yes	no	yes	yes	no

Pottery fragments were dispersed throughout the site, but unfortunately, information about their specific locations is not available.

Fauna samples were collected from all excavated units using various methods, including selected recovery from sieves, direct recovery from excavated units and/or association with funerary structures (with the anatomical position of the pieces documented in the field); coupled with concretion samples; and in total sediment samples. The systematization of these different sampling methods lacks precise information, and there is not always a clear differentiation between pickling and sieve samples.

For the zooarchaeological analysis, we focused on samples taken from ten units, three from Area A, six from Area B, and one from the Profile. Additionally, we incorporated data from previous analysis performed by André Rosa and published in DeBlasis et al. [13] for square 113/95 in Area A (Fig 2).

We studied Galheta IV’s documents and archaeological collections (fauna, human burials and pottery) at *Grupo de Pesquisa em Educação Patrimonial e Arqueologia*, *Universidade do Sul de Santa Catarina* (GRUPEP/UNISUL), Tubarão, Brazil. Permission to transport samples to France, where the isotopic analyses were performed, was granted by the *Instituto do Patrimônio Histórico e Artístico Nacional* (IPHAN) from the Brazilian government (Process #01510.000291/2020-09). All samples submitted to partially or fully destructive analysis were measured and photographed. All human teeth were submitted to microCT scanning to get morphological features based on 3D reconstruction in *Centre Inter-universitaire de Recherche et d’Ingénierie des Matériaux* (CIRIMAT—UMR CNRS 5085).

### Radiocarbon dating

Bone (Burials 2 and 9) and dentine (Burial unknown 2) samples (⁓150–500 mg) were sent to the Keck Carbon Cycle Accelerator Mass Spectrometry Facility (KCCAMS), in the Department of Earth System Science, University of California, Irvine, USA. Samples were prepared for collagen following the standard procedure at UC Irvine [56]. Radiocarbon Accelerator Mass Spectrometer (AMS) measurements were made with a National Electrostatics Corporation 0.5 MV 1.5SDH-1 Pelletron machine with a 60-sample modified MCSNICS ion source [56], on graphitized CO_2_ from ⁓2 mg of collagen. Samples were analyzed with oxalic acid standards (OX1), plus known age bone standards, modern (19^th^ century cow) and blanks that have no detectable amount of radiocarbon (Beaufort Sea whale, 60–70 kyr) that were prepared in the same manner as the unknowns.

In addition, two bones from Burials 4 and 6 were previously dated in the scope of the Jê Landscapes of Southern Brazil Project, at the Center for Applied Isotope Studies, University of Georgia, USA, and are published in this paper for the first time. These two samples were prepared with similar method of the ones described above, but measured using the CAIS 0.5 MeV Accelerator Mass Spectrometer and the sample ratios were compared to the ratio measured from Oxalic Acid II (NBS SRM 4990C).

All radiocarbon dates were calibrated using the online 4.4 version of OxCal [57], including the ones provided in this study and the ones from the literature. For the human bone calibrations, the marine curve Marine20 [58] and the atmospheric curve SHCal20 [59] were used (50/50% each, considering a mixed diet between marine and C_3_ consumption expected for shellmounds populations as quantified by Scheel-Ybert et al [60]). To account for the marine reservoir effect, which carbon is incorporated in to bone tissues from the consumption of marine resources [61], we calculate a ΔR_mean_ of -87±38 ^14^C years [62, 63]. For the fur seal bone, which was previously dated [13], only the marine curve and reservoir effect was considered. The fur seal (*Arctocephalus* sp.) is a migratory species, which may influence the reservoir effect and the result should be interpreted with caution. Conventional and calibrated dates are in Before Present (BP) time scale.

### Zooarchaeology analysis

Anatomical and taxonomic identifications of bones, teeth, shells and otoliths were made according to the zooarchaeological reference collections of GRUPEP/UNISUL and *Museu de Arqueologia e Etnologia* of *Universidade de São Paulo* (MAE/USP). NISP (Number of Identified Specimens) and MNI (Minimum Number of Individuals) were estimated [64–67]. The MNI estimates in this research were developed from the frequency of skeletal parts, while for the Otariidae (sea lions) the MNI was calculated from the skeletal frequency and also age estimates. Macroscopic thermic alterations and other types of observable modifications were also considered [64–68].

The X^2^ (or Chi^2^) test was used to test the randomness of the distribution of zooarchaeological remains in the different areas and units studied in Galheta IV in order to measure the chances of the distribution of *taxa* being random. The data used for the analyses were the NISP and MNI results of identified taxonomic families, where the minimum accepted probability of error (p) is 0.05 [69]. The software used to calculate the data was PAST [70].

### Carbon and nitrogen isotope analysis

Collagen was extracted at the Géosciences Environnement Toulouse, Observatoire Midi Pyrénées (GET/OMP) in Toulouse, France. Specimens were cleaned with a dental drill using diamond tipped burrs and approximately 500 mg of bone and tooth fragments were used. Samples were demineralized with 0.5 N hydrochloric acid (HCl) at 4°C. Demineralization time depended on the preservation of each sample, and took between a few days to a week. Specimens were treated with 0.1 N sodium hydroxide (NaOH) for 30 minutes, rinsed with MilliQ water until neutral. Then placed in pH 3 solution and solubilized on a block heated at 75°C for 24 hours. The solution was then filtered with a 5–8μm filter (Ezee filters), and then ultrafiltered using >30 kDa molecular filters to remove small and damaged collagen molecules. The fraction >30 kDa was transferred to glass tubes and then lyophilized. Collagen (⁓0.3–0.5 mg) was weighed into tin capsules and measured in duplicate at Iso-Analytical Ltd. (Crewe, United Kingdom). The stable isotopes of carbon and nitrogen were analyzed as the ratio of the heavier isotope to the lighter isotope (^13^C/^12^C or ^15^N/^14^N) and are reported as δ values in parts per 1,000 or “per mil” (‰) relative to internationally defined standards for carbon (Vienna Pee Dee Belemnite, VPDB) and nitrogen (Ambient Inhalable Reservoir, AIR) [71]. The analytical error (1 SD) was determined to be 0.1‰ for both δ^13^C and δ^15^N results according to the standards of Iso-Analytical Ltd. We have multiple δ^13^C and δ^15^N results for certain individuals, as these measurements were taken at a later stage in the sample preparation process employed for radiocarbon dating.

### Strontium isotope analysis

Strontium samples were prepared following the protocol of Deniel & Pin [72] and described by Richards and colleagues [73]. This involved suspending 0.5 cm of Sr-spec 50–100μm resin in Milli-Q water, which was previously cleaned in two rounds also using Milli-Q water. The column was conditioned with 3 ml of 3N HNO_3_, before sample loading. The matrix was eluted with 3N nitric acid (HNO_3_), the strontium was eluted/recovered with 5 ml of Milli-Q water. The values were normalized using the reference material SRM 987 (^87^Sr/^86^Sr = 0.70924) that was measured with samples in each measurement session. For each batch of samples prepared, at least one blank and an international internal standard sample (NIST SRM 1400, with bone ash) were added, as a reference to check for possible contamination and control of the protocol. The SD varied between ± 0.000004 to ± 0.00007. Strontium isotopic ratios were obtained at the GET/OMP in Toulouse, France on a MC-ICP-MS (Neptune Plus) and a NuPlasma 500 at the École Normale Supérieure (ENS) in Lyon, France.

Trace element concentrations were obtained for most of the samples analyzed for strontium isotopic ratio analysis. We used a Thermo Fisher ICAP Triple Quadrupole (TQ-ICP-MS) from the GET/OMP laboratory. Aliquots of 20 to 50 μL collected after digestion of the samples were diluted in a solution containing 0.37 N HNO_3_ and a standard solution of Indium (In) and Rhenium (Re) with known concentrations of ⁓2 ppb. Thus, the concentrations of the elements of interest (Ca, Sr, Ba, Fe, Zn, Cu, Al, Mn, Mg and P) n a given sample are calculated based on the standard internal and external samples.

### Ceramics analysis

Itararé-Taquara ceramics are characterized by small, thin-walled vessels. The shapes vary between cylindrical, spherical, conical, or ovaloid, and the contours are simple or inflected. The external surface can be smoothed, smudged, colored (red or dark slip), or decorated (incised, punctated, fingernail marks, stamped motifs, or basketry imprints) [45, 74–77].

The ceramic fragments were classified according to the main attributes described in previous researches on the subject [78–83]. These include attributes related to the manufacture, external surface, and shape of the vessels, which provide information about the technological and cultural aspects involved in the production of the vessels [84, 85]. The attributes analyzed were: type of fragment (class), the addition of temper, manufacturing method, firing atmosphere, surface treatment type, morphology (rim and base), and functional/use aspects. The vessel forms were reconstructed from drawings of the rim profiles and based on previous research [45, 74–77]. Functional and use aspects were inferred from vessel forms, size and capacity, accessibility of contents, and transportability [78, 79], but also from traces of alteration by human activities, such as cooking (carbonized food deposits) [80].

### FTIR on heated bones

Evidence of heating were frequently described both for the human and faunal bones recovered from Galheta IV site. For human bones, the identification of heating has been attributed to cremations at Galheta IV, a funerary practice that characterizes Southern *proto*-*Jê* populations [32, 33]. However, no other evidence for cremations have been described at the site, despite the dark coloration identified in some human bones.

Color charts have been frequently used as a macroscopic proxy for the identification of burning in bone fragments, however, taphonomic processes can also induce similar changes in bone coloration [86]. To investigate the presence of heated bones at Galheta IV, we sampled human and faunal bones according to the visual criteria commonly associated with heating, such as color and shape, and analyzed them by Fourier Transformed Infrared Spectroscopy (FTIR). Selected bones were classified into five categories: 1) bones with natural color—apparently without thermal changes; 2) bones with natural color and changes in shape, such as cracking and warping; 3) brown bones—slightly burnt; 4) black-colored bones—charred; and 5) white bones—calcined.

Bone fragments of each category were grinded using an agate mortar and pestle. Analyses of the bone powder were done by Attenuated Total Reflectance (ATR) with a Cary 670 series equipment and Resolutions Pro software from Agilent Technologies. The spectra were collected between 400 and 4000 cm-1, with 4 cm-1 resolution, at the Institute for Archaeological Sciences of the University of Tübingen. To determine the approximate heating temperatures, all spectra were compared with data from the literature [87–89]. Peak measurements, to calculate the crystallinity index (CI) or infrared splitting factor (IRSP) [86, 87, 90–93], were done using Essential FTIR software at the Microarchaeology Laboratory of the University of São Paulo. Both CI and IRSP are commonly used to determine heating and/or taphonomic processes affecting bone assemblages.

## Results

### Radiocarbon dating

**Fig 3:**
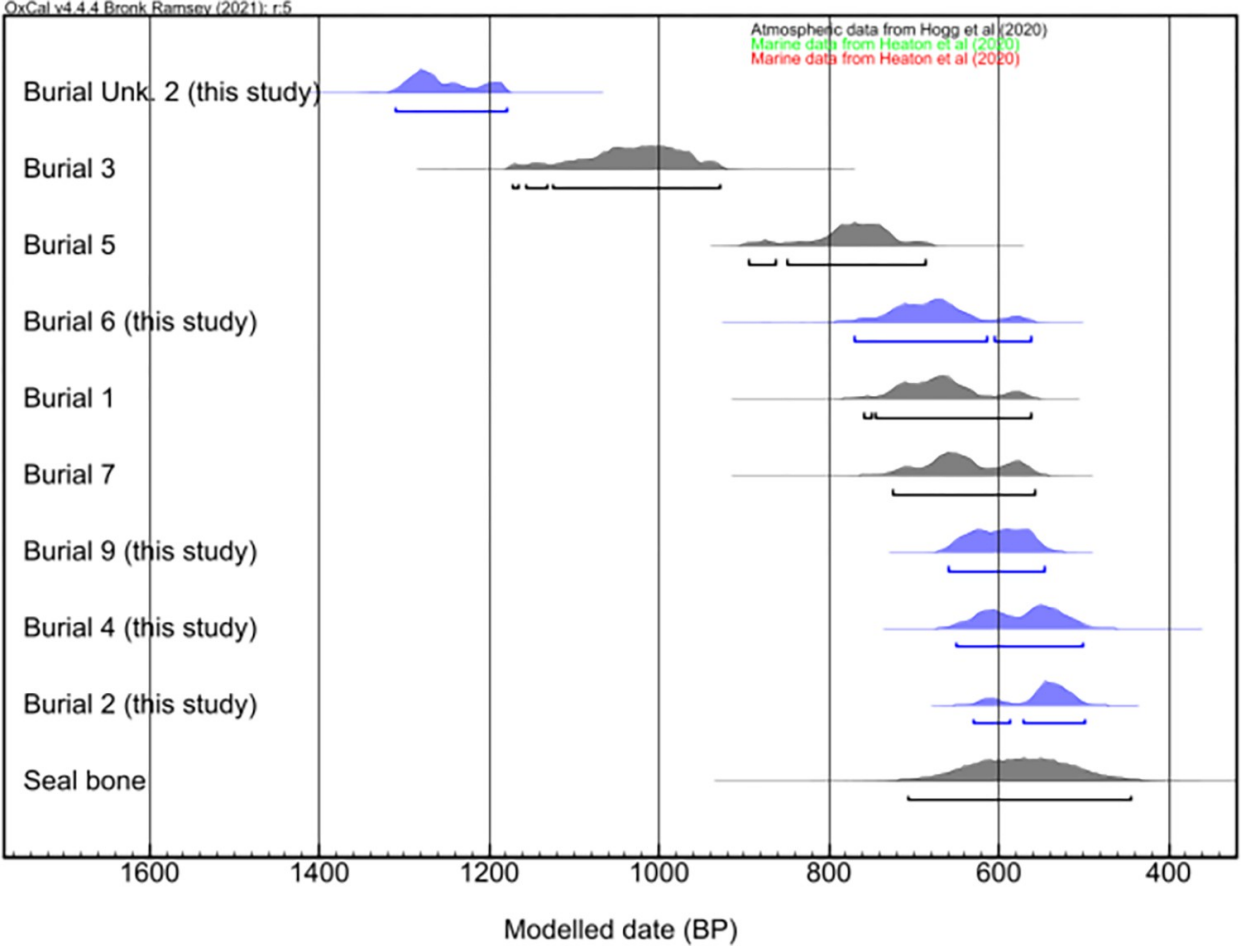
Calibrated radiocarbon dates for humans’ burials of Galheta IV archaeological site. In blue the new dates obtained in this study.

Five new radiocarbon dates on human remains are reported here (Table 2, Fig 3). One of these dates (Burial unknown 2, 1305–1180 years cal BP) is highly significant as it suggests that the site was used approximately two centuries earlier than previously believed. Nevertheless, it is essential to approach this finding with caution since the tooth used for dating was found in the fauna sample bags, detached from a specific funerary context.

**Table 2:** Radiocarbon dates for Galheta IV archaeological site.

Sample	Material	Lab ID	^14^C age (BP)	±	^14^C age calibrated (BP) (95.4%)	Reference
Human—Burial 1	Bone	Beta 211734	980	40	744–562	Farias & Deblasis, 2007
Human—Burial 2	Bone (cranium)	UCIAMS 263362	805	15	629–498	This study
Human—Burial 3	Bone	Beta 280010	1360	40	1173–931	Farias & DeBlasis, 2007
Human—Burial 4	Bone (rib)	UGAMS 30089	830	43	650–502	This study
Human—Burial 5	Bone (petrous)	MAMS-40646	1105	17	893–685	Ferraz et al, 2023
Human—Burial 6	Bone (rib)	UGAMS 30090	990	44	769–563	This study
Human—Burial 7	Bone	Beta 280012	950	40	725–555	Farias & DeBlasis, 2007
Human—Burial 9	Bone (femur)	UCIAMS 263363	885	15	658–546	This study
Human—Burial unknown 2	Dentine (molar)	UCIAMS 263364	1575	15	1305–1180	This study
Seal (*Arctocephalus* sp. 112/93, Level 3)	Bone	Beta 280011	1070	40	707–443	Farias & DeBlasis, 2007

Currently, we have obtained a series of ten radiocarbon dates for Galheta IV, with nine of these from human remains and one from an aquatic mammal bone. These results demonstrate that there were distinct chronological periods. Two radiocarbon dates are associated with the oldest burials, dating between 1305–1180 years cal BP (Burial unknown 2) and 1173–931 years cal BP (Burial 3b). After, a period encompassing the remaining burials, ranging from ca. 900 to 500 years cal BP, suggesting a contemporaneous and continuous visitation during the later period.

### Zooarchaeology

A total of 21,444 zooarchaeological fragments were analyzed, corresponding to 37 families, 33 genera, and 21 species. More than 95% of the identified specimens were fragments of bones, teeth, and otoliths and only 3.7% were shell fragments. MNI results showed a predominance of Teleostei (bony fishes), followed respectively by gastropoda (snails), birds, mammalia (mammals), elasmobranchii (sharks and rays), crustacea (crabs), and chelonia (turtles).

The predominant vertebrate *taxon* is the *Trachinotus* sp. (Carangidae—pompano fish), which is a pelagic fish frequently found in shoals and reported in the region mainly in winter, in the open sea, near to the rocky shores [94–96]. The amount of *Trachinotus* sp. estimated for the site represents 60% of the MNI between the ichthyofauna, and 47% of the MNI of all *taxa* identified.

Other predominant vertebrate *taxa* are the fishes of the family Sciaenidae (mainly *Micropogonias furnieri*—whitemouth croaker. MNI = 13.5%), albatrosses (*Thalassarche* sp. MNI = 7%), marine catfish (Ariidae, MNI = 7%), snook fish (*Centropomus* sp. MNI = 4.6%), mullet fish (*Mugil* sp. MNI = 4%), fur seal (Otariidae, *Arctocephalus australis*. MNI = 3.2%) and penguins (*Spheniscus magellanicus*. MNI = 1.8%). The other 23 *taxa* correspond to 10.5% of the MNI. Almost all of the identified animals belong to a marine or estuarine environment (approximately 98%) while terrestrial animals like wild pigs (*Tayassu* sp.), deer (Cervidae) and tortoises (Emydidae) occur in low frequencies (see all in S1 Table).

**Fig 4:**
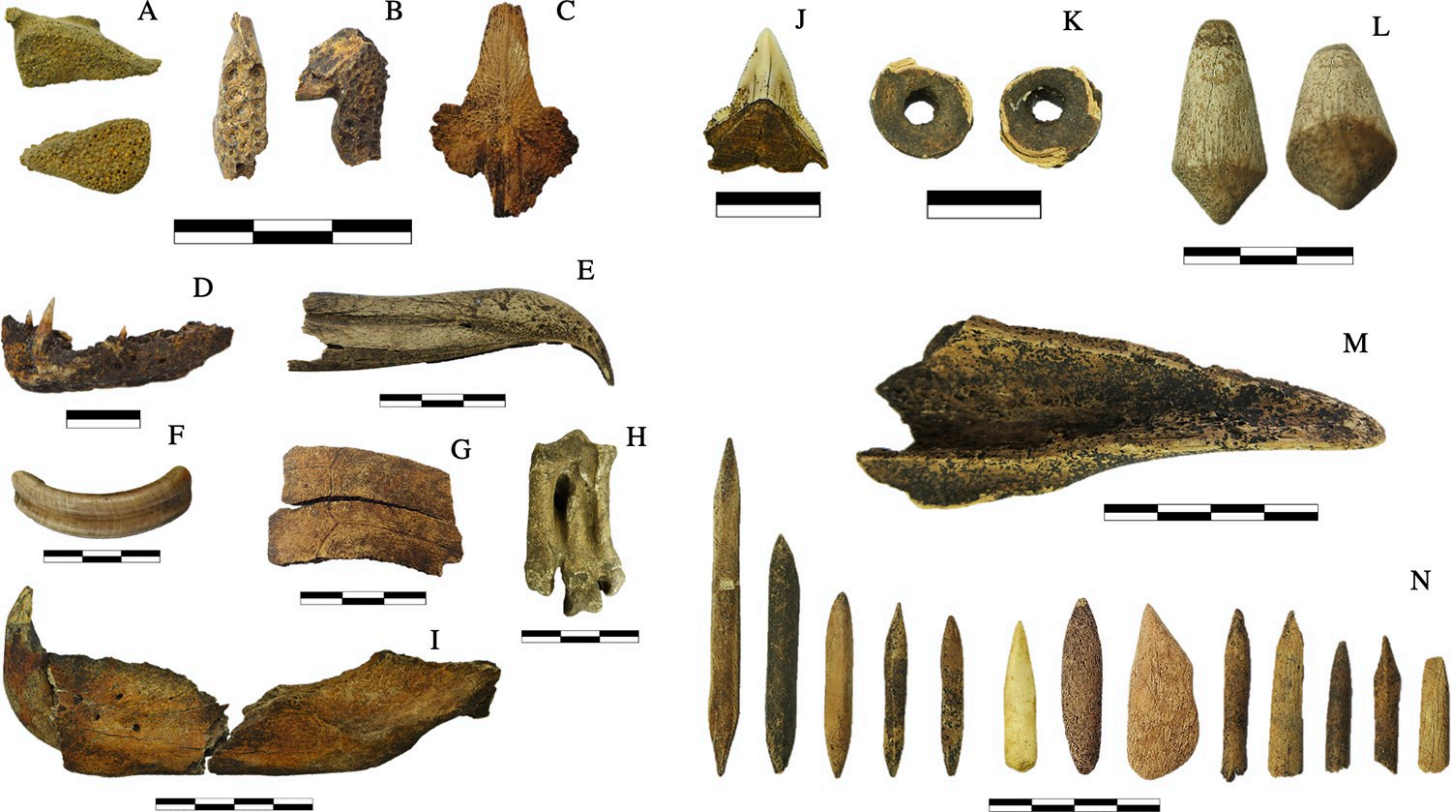
Diagnostic bones and teeth used for taxonomic identification, and tools and adornments made from faunal remains. A: *Trachinotus* sp. B: *Archosargus probatocephalus*. C: *Genidens barbus* D: *Hoplias malabricus*. E: *Thalassarche* sp. F: *Hydrochoerus hydrochaeris* G: *Trachemys* sp. H: *Spheniscus magellanicus* I: *Arctocephalus australis* J: tooth of *Carcharodon carcharias* polished and drilled twice at the root. K: Elasmobranchii vertebrae perforated. L: Cervidae horn retainer. M: bone tool made of mammal long bone. N: one-sided or two-sided bone tips made from bird and mammal long bones. Note: Own elaboration.

The fauna bones and teeth were used to make tools and adornments and a total of 35 pieces were found at the site (Fig 4). These included: one- or two-sided bone tips made from bird and mammal long bones (probably fish hooks), fusiform-shaped artifacts made from mammalian bone, adornments and/or tools [97, 98] made from elasmobranch vertebrae and teeth (mostly found in the funerary contexts), and an artifact made from deer antler.

The NISP and MNI data were grouped into taxonomic families and then statistically analyzed using X^2^ and clustering tests between the different analyzed contexts, with the objective of measuring the chances of the distribution of *taxa* being random, and then establishing comparative intra-site interpretations (detailed description in S1 File). The test results from the data of these two variables (NISP and MNI) showed differences. Thus, statistically distinct areas were identified because of their zooarchaeological content (p<0.05). There was an area at the southeast of the site (A Area, unit 112/93) with a large accumulation of fauna remains, in opposition to the units where the burials were found. Besides the material being more abundant in this southeast A Area, we also have the presence of larger animals’ bones and teeth, especially cetaceans and pinnipeds, as well as terrestrial mammals, such as pigs and deer.

### δ^13^C and δ^15^N isotopic results

**Fig 5:**
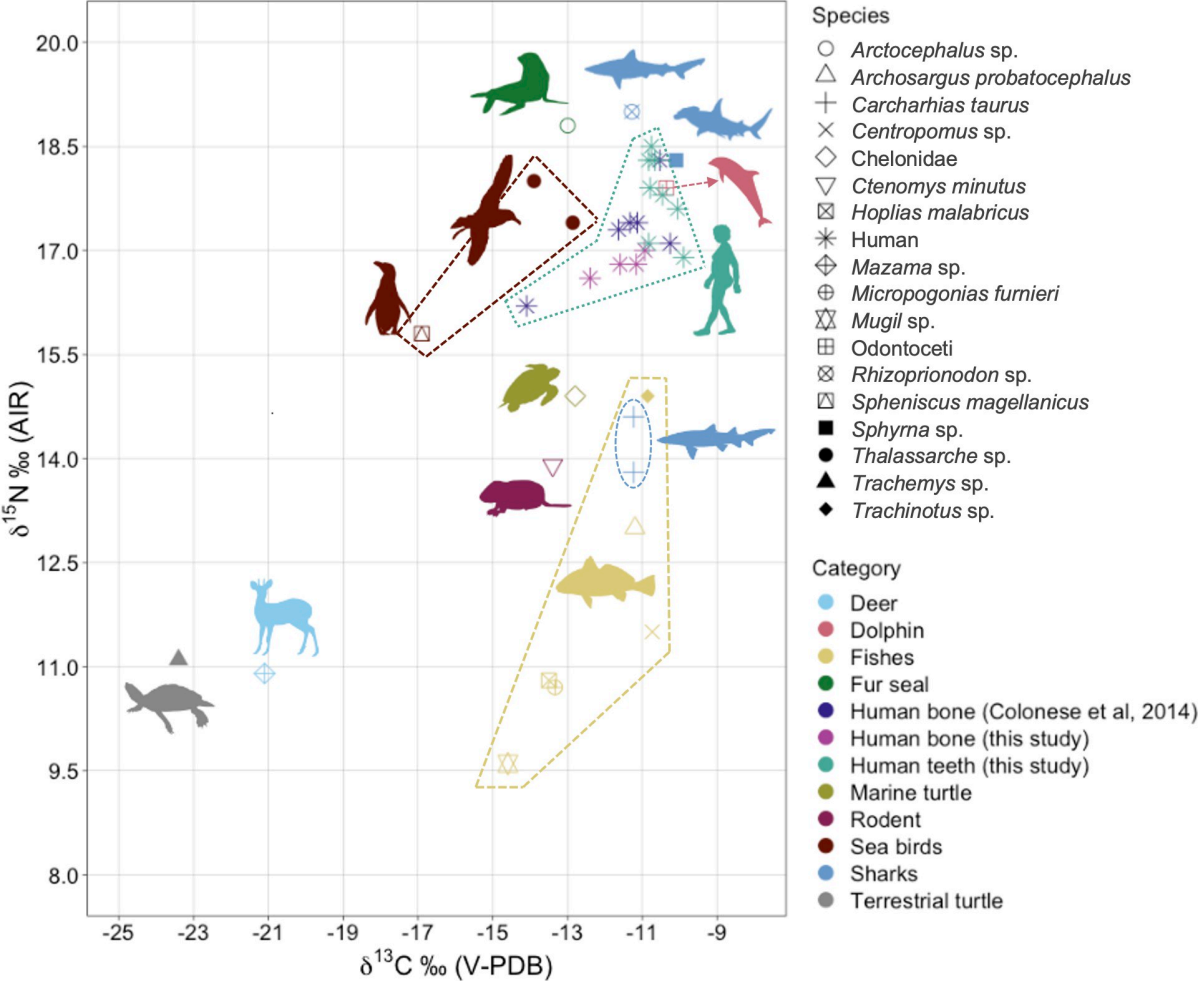
δ^13^C and δ^15^N results for Galheta IV humans and fauna samples (n = 38).

**Fig 6:**
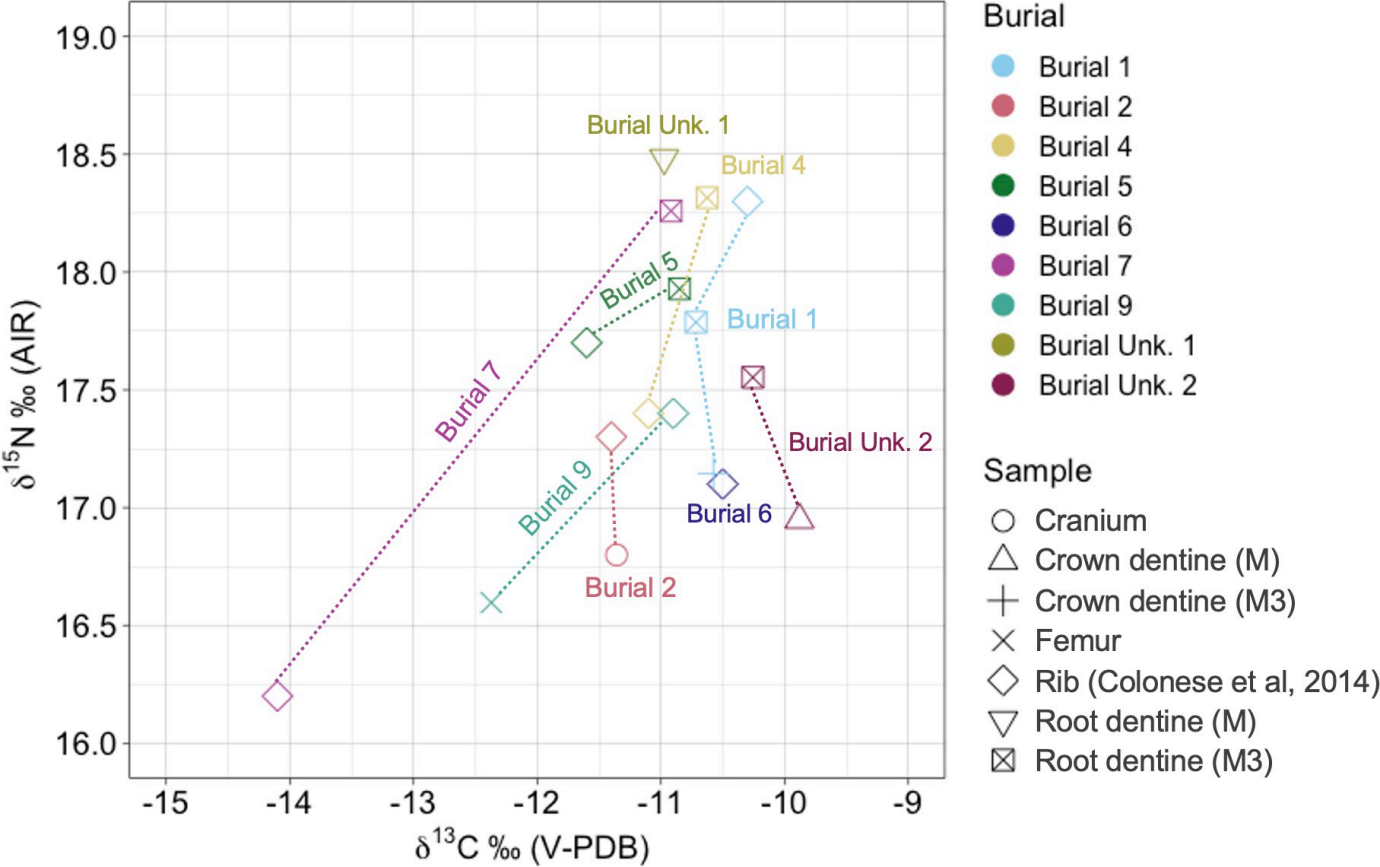
δ^13^C and δ^15^N results for Galheta IV humans’ bones and dentine samples (n = 18).

The bulk collagen δ^13^C and δ^15^N isotopic results are plotted with previously published data from humans’ bones [28]. Additionally, we analyzed human dentine (crown and/or tooth root), and zooarchaeological bone samples (Figs 5 and 6). Collagen was successfully extracted for 30 out 32 fauna and human samples and with yields above >1%, C:N ratios between 3.1 to 3.4.

The δ^15^N values of the fishes are important to interpret those populations’ diets. Certain sharks (*Rhizoprionodon* sp. and *Sphyrna* sp.) showed high nitrogen isotopic ratios (19.0‰ and 18.4‰), while *Mugil* sp. (mullet) had the lowest (9.6‰). Among the most abundant *taxa* in the zooarchaeological assemblage, *Trachinotus* sp. (pompano) displayed δ^15^N values similar to those of *Carcharias taurus* (tiger shark), at 15.0‰, 14.0‰, and 14.7‰, respectively. Interestingly, the freshwater fish *Hoplia malabricus* displayed isotopic ratios similar to those expected for marine fishes (δ^13^C = -13.5‰ and δ^15^N = 10.8‰), suggesting that these species might have moved between rivers and estuarine areas.

Most of the δ^15^N results of the Galheta IV’s fishes correlates with the expected trophic levels of the same species according to the FishBase dataset [99]. We observed only two exceptions on *Carcharhias taurus* and *H*. *malabricus*, which present lower δ^15^N than expected. However, those variations can be related to the specimens’ ages and environments.

The terrestrial animals (*Mazama* sp. and *Trachemys* sp.) exhibited typical C_3_ dietary values. Seabirds exhibited δ^13^C isotopic ratios ranging from -12.5‰ to -13.9‰, and δ^15^N ratios from 17.0‰ to 18.0‰, slightly lower than those of seals (*Arctocephalus* sp.). The *Ctenomys minutus* had a δ^13^C value of -13.4‰ and a δ^15^N value of 13.9‰, indicating that rodents of this species likely consumed both marine and C_3_ resources.

The human isotopic results of our study agree with the previously published work [28] and show that the main source of protein in this population comes from marine resources. The δ^13^C and δ^15^N ratios obtained from the bones of Burials 4 and 7 were lower when we compared with teeth root values. In contrast, variations observed in the ratios of other individuals do not appear to be significant, as they fall within the standard deviation. For Burials 4 and 7, we can consider variations in the diet of these individuals between childhood and adolescence, possibly involving a greater consumption of C_3_ food sources in comparison to the other individuals buried in Galheta IV, who predominantly relied on marine resources.

### Strontium isotope ratios of tooth enamel (^87^Sr/^86^Sr)

Due to the incomplete nature of the skeletal remains, not all individuals had teeth available for analysis. Thus, strontium isotope analysis was conducted on eight enamel samples, corresponding to seven individuals. The upper part of the third molar (M3) crown was sampled for Burials 1, 4, 5, 7, and Burial unknown 2, the canine was sampled for Burial unknown 2, the first pre-molar (PM1) was sampled for Burial 3b, and a fragment of an unknown molar tooth was sampled for Burial unknown 1.

In Brazil, there is a lack of a comprehensive strontium isotope database to establish a local baseline for available strontium in the geological context. Therefore, we analyzed the archaeological fauna to aid in interpreting the origin of both local and non-local individuals at Galheta IV. Although, due to the scarcity of fauna teeth, particularly from terrestrial species, we conducted experiments on bones, shells, and samples of enamel and dentine. The analysis of trace elements revealed diagenesis in many of these samples, often resulting in elevated concentrations of Sr, Fe, Al, and Mn. Even some enamel samples showed evidence of diagenesis. Consequently, only eight carefully selected fauna samples were successfully analyzed to establish local values and create a baseline for ^87^Sr/^86^Sr.

**Fig 7:**
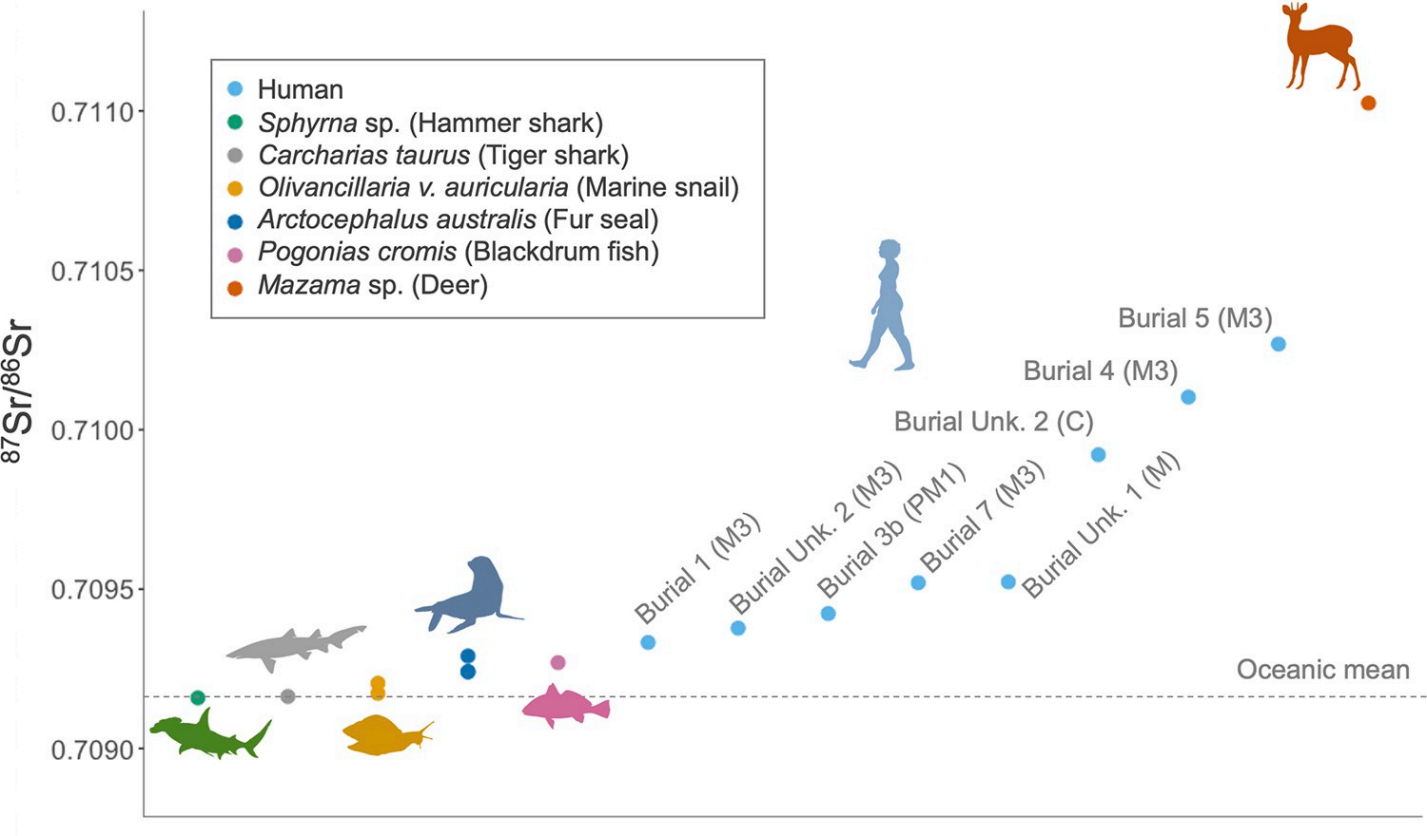
Strontium isotopic ratios of humans and fauna enamel samples of Galheta IV (n = 15).

All marine fauna exhibited ^87^Sr/^86^Sr values close to those of strontium dissolved in seawater (^87^Sr/^86^Sr_ocean_ = 0.7092, [29, 31]). An enamel sample from *Mazama* sp. (deer), had a value of 0.71078. Among humans, the average value was 0.70968, indicating that while the primary source of strontium for these individuals came from the ocean, they also consumed resources from terrestrial environment. Burials 4, 5, and unknown 2 exhibited slightly higher values, 0.71010, 0.71027, and 0.70992, respectively. The Burial unknown 2 had on ^87^Sr/^86^Sr variation among the third molar and the canine, where the canine exhibits the higher strontium isotopic ratios (Fig 7).

### Ceramics

Out of a total of 190 ceramic fragments recovered from the excavations at Galheta IV, 131 were analyzed (68.9%). The remaining fragments were not analyzed due to their high fragmentation and small size (<2 cm). Overall, the ceramic paste is homogeneous and has a non-plastic inclusion of fine minerals (quartz sands) (74.1%), followed by coarse minerals (quartz) that may have been added to the paste (25.9%). Most of the fragments indicate that the ceramic vessels were produced by coiling (62.6%), while the others were produced by mixed forming techniques, such as paddling/beating [79–81]. Mixed forming techniques can be identified by the absence of coil negatives, irregular fractures, and fragments of low thickness. The use of this technique produces vessels with thin walls and rounded bases, which are characteristic of the Itararé-Taquara ceramics. The fragments have a thickness of between 3 and 10 mm, with the highest percentage between 4 and 6 mm (76.3%). The majority of the fragments also indicate the prevalence of a total reduced atmosphere (66.4%), followed by a partially reduced atmosphere (17.6%). Most of the fragments are externally and internally smoothed (63.4% and 67.2%, respectively). There were also fragments with plastic decoration (19.8%) and colored with a thin dark slip (14.5%). Rare were the fragments colored with a thin red slip or smudged (0.7% each).

**Fig 8:**
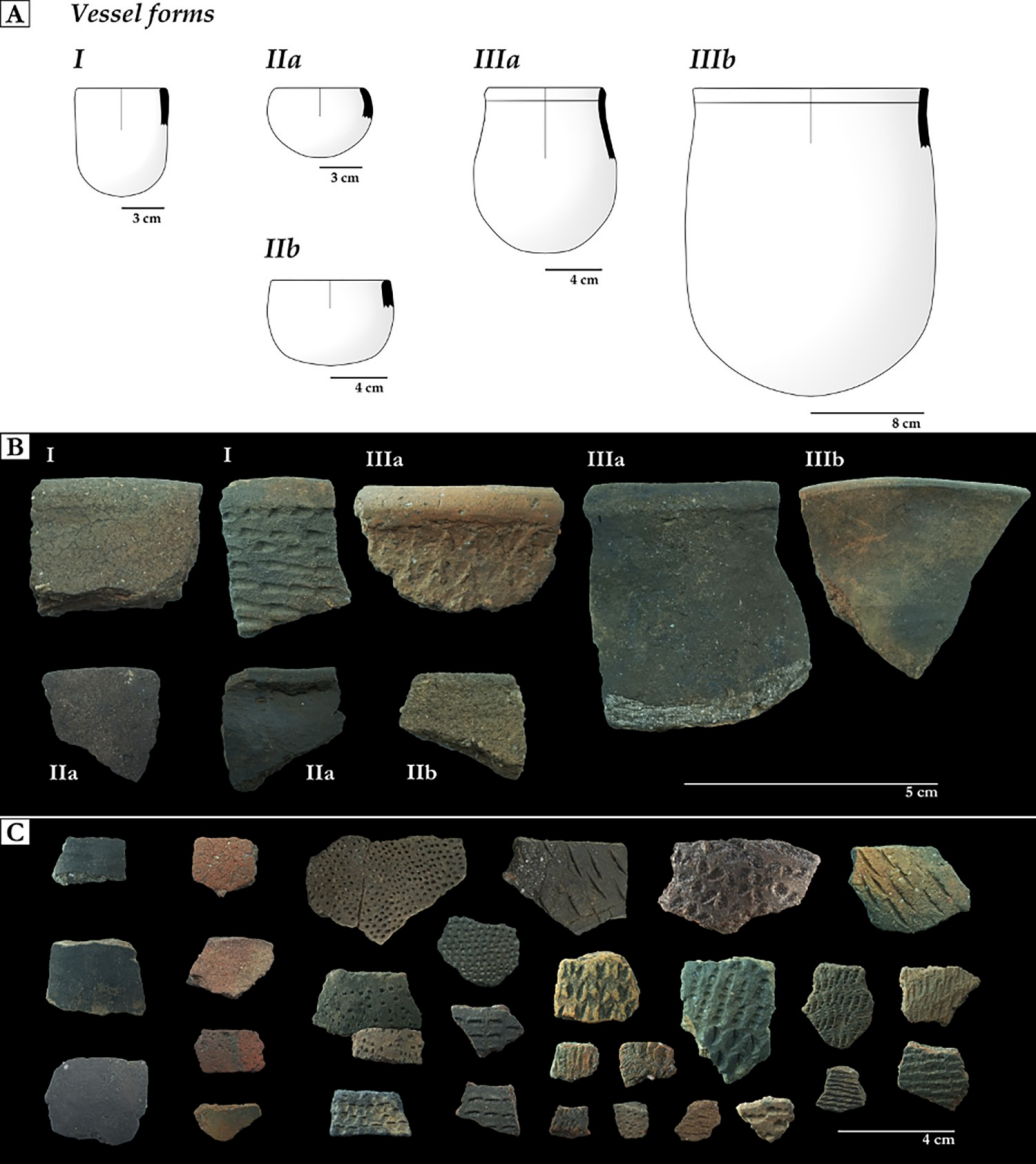
Ceramics from Galheta IV. A: the reconstructed vessel forms for Galheta IV. B: examples of external surface treatments recorded on the reconstructed forms for Galheta IV. C: the general set of external surface treatments recorded: smudged or with thin dark slip, thin red slip and plastic decoration (from left to right). Note: Own elaboration.

As for the shape of the bases (n = 15), the concave shape predominated (80%), while only one was flat. The other two bases were too small to read the shape. Three vessel forms were reconstructed from the collected rims (n = 18). Form I (16.7%) was simple, cylindrical, and not-constricted, with a diameter ranging from 6 to 20 cm. The external surface is smoothed, smudged, or punctated. Form II (44.4%) had a simple contour with spherical (IIa) or cylindrical (IIb) shapes, diameter range between 6–12 cm, with smoothed as the predominant external surface treatment. Fragments with a thin dark slip or smudged outer surface were rare. Considering the general characteristics of size and shape, Forms I and II were related to vessels for individual use, such as cups or bowls [79]. Form III vessels (38.9%) had an inflected contour, slightly restricted, with ovoid (IIIa) or cylindrical (IIIb) shapes and a thickened rim. The external surface generally has a thin dark slip but also occurs with smoothed or finger-pinched surface treatments. Form III vessels are related to small jars, with a diameter ranging from between 6 to 16 cm (Fig 8).

### FTIR on heated bones

A total of 14 samples, including 8 human and 6 bird long bones, were analyzed by FTIR-ATR to investigate heating. The results revealed different levels of burning, which were sometimes coincident with differences in coloration (e.g. brown, black, and white). The heating temperatures ranged from approximately 300°C to at least 800°C.

Fauna bones that macroscopically appeared unaltered, were heated at low temperature, between 300–400°C (Table 3). This color was observed in 83.3% (n = 17,974) of the fauna analyzed. Bones with natural color and cracks on their surface were also heated at low temperature, above 400°C, and corresponded to 0.1% (n = 11) of the material analyzed. Bones with shades of brown had a burning temperature above 600°C, and represented 2.4% (n = 504) of the analyzed fauna. Black bones, which sum 6.8% (n = 1,449) of the analyzed material, also had an estimated heating temperature above 600°C. White colored bones were heated at temperatures above 800°C (calcination), and represent 7.0% (n = 1,506) of the analyzed fauna at the site.

**Table 3:** Bones analyzed by FTIR-ATR at Galheta IV.

Sample	Material	Description	CI/ IRSF	Temperature estimation (°C)	Frequency on the site
Human—Burial 1	Femur	Black color/charred in the bone surface and shape modifications	4,72*	>600	NA
Human—Burial 2	Cranium	No visible modifications	4,14	>600	NA
Human—Burial 3b	Femur	Black color/charred	4,44**	>800	NA
Human—Burial 4	Femur	Brown color	4,53	>500	NA
Human—Burial 5	Cranium	Brown color	4,30	>400	NA
Human—Burial 6	Tibia	Brown color	4,56	>400	NA
Human—Burial 7	Pelvis	Brown color	4,34	>400	NA
Human—Burial 9	Tibia	Brown color	4,51	>400	NA
Fauna 1	Bird long bone	No visible modifications	3,78	>300	83.8%
Fauna 2	Bird long bone	Natural color, shape modifications	4,06	>400	0.1%
Fauna 3	Bird long bone	Brown color	4,33	>600	2.4%
Fauna 4	Bird long bone	Black color/charred	4,15	>600	6.8%
Fauna 5a	Bird long bone	White color/calcinated	5,50**	>800	7.0%
Fauna 5b	Bird long bone	White color/calcinated	5,42**	>800	

CI = crystallinity index; IRSF = infrared splitting factor

* = low peak at ~630 cm^-1^

** = peak at ~630 cm^-1^.

**Fig 9:**
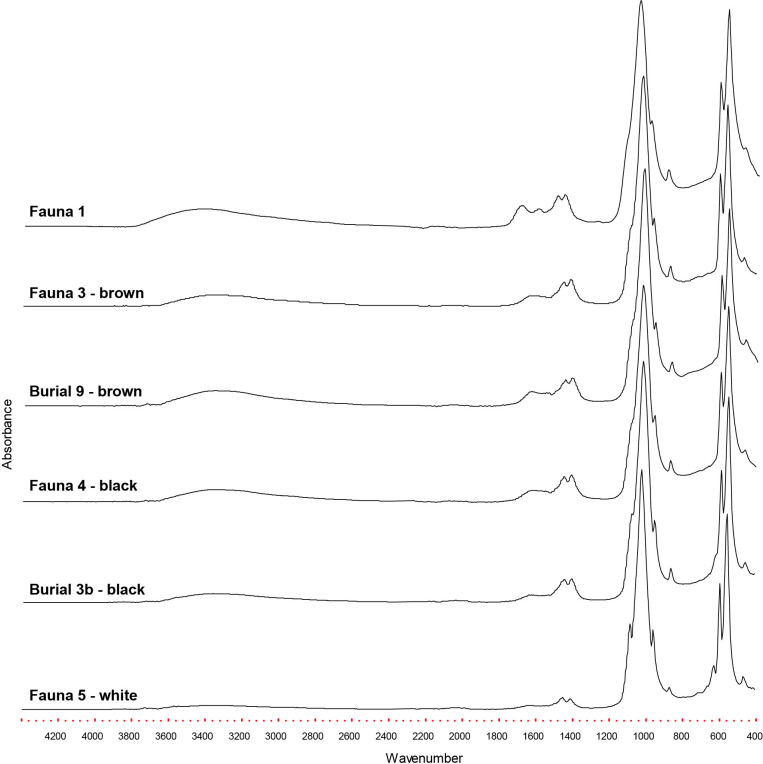
FTIR-ATR spectra of faunal and human bones from Galheta IV, representative of the different colors and heating temperatures.

FTIR-ATR analysis of human bones indicates thermal alteration at low to high temperatures. Specific bones from burials 4, 5, 6, 7, and 9 were exposed to low temperatures, above 400°C. Burials 1, 2 and 3b were heated at above 600°C (Burials 1 and 2), and even above 800°C (Burial 3b), which correlates with the observed macroscopic alterations (Table 3, Fig 9).

## Discussion

### Movements to the coast and along the coast

Our analysis indicates that the occupation of Galheta IV began between 1305 and 1180 years cal BP which represents an earlier occupation than previously documented for the settlement (1173–931 years cal BP [13]). The oldest dates recorded for the presence of Itararé-Taquara ceramics were found at the Enseada I site in the north of Santa Catarina (1390±40 years BP, 1180–938 years cal BP, [100]), and at the Praia das Laranjeiras II site along the central coast (1340±30 years BP, 1112–922 years cal BP, Beta 426439, [101]). Thus, the Galheta IV chronology could be pushed back by several decades from the chronological boundaries (ca. 1200–900 years cal BP) proposed by Toso et al [18] for the earliest evidence of the Itararé-Taquara pottery along the Santa Catarina coast (S2 Fig).

The occupation of Galheta IV lasted for at least 800 years without any evidence of abandonment. Most of the individuals were buried from around 900 to 500 years cal BP. Two individuals presented dates between 1300 to 900 years cal BP. In this sense, it seems that there were recurrent visitations to the site during the last 300 years of its occupation. The 190 ceramic fragments at Galheta IV were found scattered throughout the site. This abundance is quite small when compared with other late Holocene sites located on the central and north coasts of Santa Catarina, such as Praia das Laranjeiras II, Tapera and Enseada I, where more than 4500 ceramic fragments were found [101–106]. Despite the smaller sample size, our detailed analysis reveals interesting patterns in the external surface treatments and vessel forms, which are related to regional differences of the Itararé-Taquara tradition [75]. The prevalent presence of smoothed, thin dark slip or smudged surfaces suggest a closer resemblance to the Itararé phase assemblages documented on the adjacent north coast of the study area, rather than those found in the highlands.

By comparing the data presented in this study for the Galheta IV pottery with data from previous research, it was possible to make correlations that may indicate areas where Galheta IV pottery has greater similarities. Vessel forms I and II, which represent almost one-third of the sample, are common in the southern part of the study area (in northeastern coast of Rio Grande do Sul state) and are associated with the Taquara phase [45, 76, 77]. Conversely, vessel form III is more common on the north coast of the study area and is associated with the Itararé phase [45, 74, 77]. These results indicate that there were movements of Itararé-Taquara ceramics along the coast, as hypothesized before based on strontium isotope variations [107–109].

**Fig 10:**
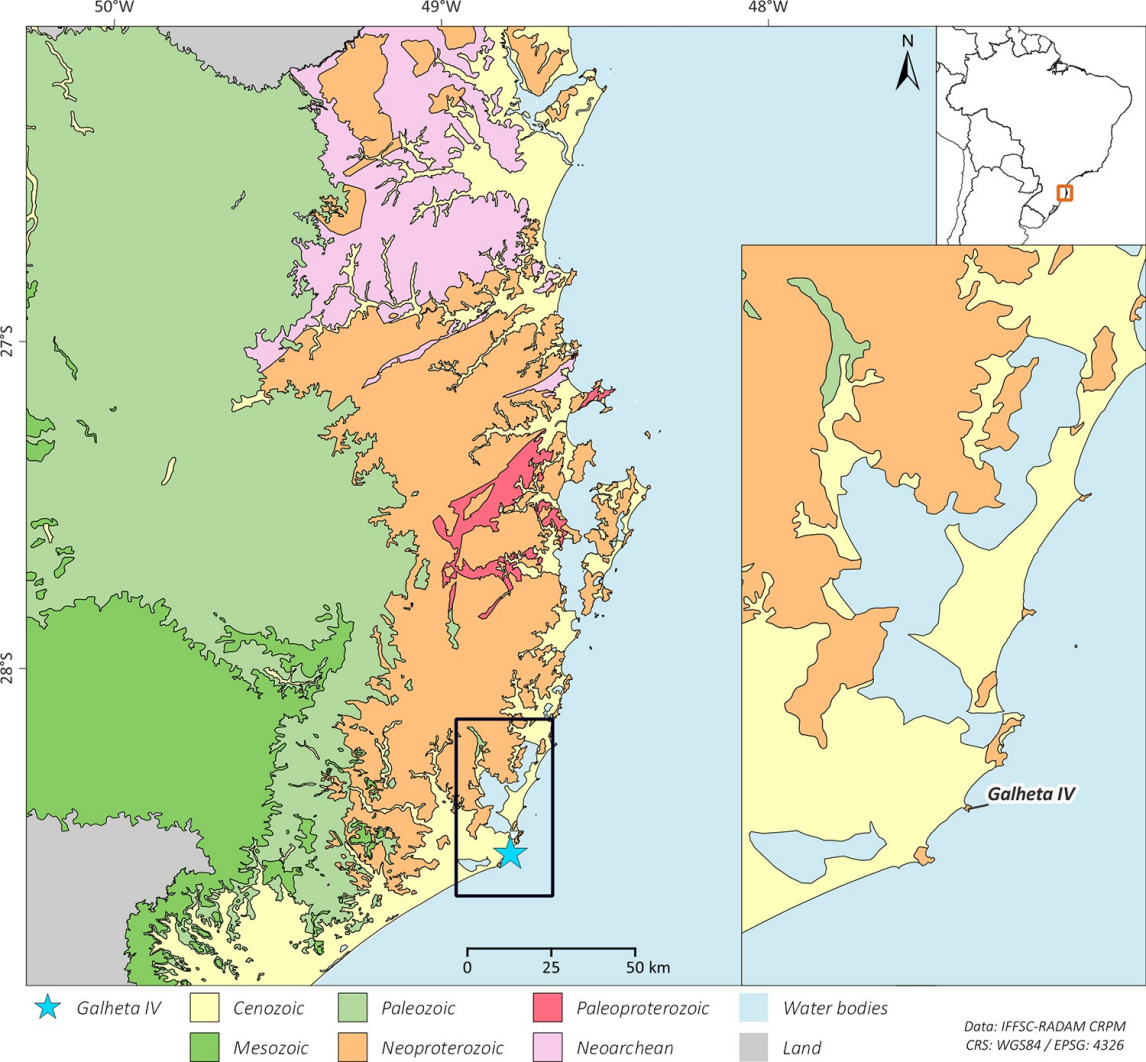
Geological map of part of southern Santa Catarina coast, with the region of the site Galheta IV magnified on the right corner. Made with free vector and raster map data. Geological units: Brazilian Geological Survey (SGB) [115]; South America Administrative Boundaries: Brazilian National Water Agency (ANA) [116]. All utilized geographical data are under the Creative Commons Attribution License (CC BY 4.0). Software: QGIS 3.28. Note: Own elaboration.

Regarding the strontium isotopes results, it is important to notice that the bedrock of the region where Galheta IV is located is formed mainly by Neoproterozoic alkaline granites with a sedimentary cover from the Cenozoic (quaternary deposits) [110]. No strontium isotope mapping is available for the area. However, some data from adjacent regions [111–113] shows that the terrestrial fauna data obtained in this research correspond to the expected bioavailable strontium isotopic ratio for the area (range from ~0.71 to 0.73). The geology of the highlands, where Southern *proto*-*Jê* occupation was mainly registered, is formed by younger Cretaceous volcanic rocks, like basalts [110], and the expected strontium isotopic ratios would be lower when compared to areas close to the coast [114] (Fig 10).

Our ^87^Sr/^86^Sr results for Galheta IV show that all the eight individuals analyzed at the site are compatible with local values. Still, some variations can be observed between individuals or over the course of the life of an individual (by comparing different teeth). These variations have two possible origins: a variable intake of terrestrial resources and/or geographical mobility. The variable intake of terrestrial resources is also supported by the carbon and nitrogen isotope data that highlight interindividual and intraindividual variations over their lifetime (see the discussion about the diet at the next section).

The individuals of the Burials 4 and 5 exhibited higher enamel ^87^Sr/^86^Sr for their third molars than the rest of the population, indicating a stronger reliance on terrestrial resources during the age of 9 to 13 years old [117]. For the individual of the Burial unknown 2, the ^87^Sr/^86^Sr of their canine enamel, formed between 6 months and 4.4 years, is higher compared to that of the third molar, indicating slight variations in the individual’s diet and/or living place during late childhood and adolescence. All other individuals seem to have relied on marine resources and/or lived directly on the coast during their adolescence.

As mentioned above, the ^87^Sr/^86^Sr of all individuals are compatible with the local bioavailable strontium ratios (both marine range or similar to the terrestrial animal). We therefore did not spot any evidence of migrations coming from more inland sites. Previous studies on strontium isotopes ratios in the north and central Santa Catarina State also showed that the presence of Itararé-Taquara pottery does not seem related to an occupation of groups from the interior [107–109]. Nevertheless, possible movements throughout the coastline are not discarded.

Despite those results, previous research identified a genetic affinity between the *sambaqui* culture and the Southern *proto*-*Jê* through an individual from the late levels of Jabuticabeira II fish mound (~1300 years cal BP) [25, 55]. However, archaeogenetic analysis of an individual from Galheta IV (Burial 5) revealed a greater affinity with individuals from the *sambaqui* Cabeçuda (~4000 to 2800 years cal BP) and the shell layer of Jabuticabeira II (~2400 years cal BP) [25].

In summary, we were able to discard the possibility of Galheta IV being a Southern *proto*-*Jê* settlement at the coast from our study. Instead, our study suggests that Galheta IV should be considered a funerary mound resulting from prolonged interactions between the both populations, *sambaqui* and the Southern *proto*-Jê. The development of new archaeogenetic studies, the increase of a database for strontium isotopes and new comparative analysis of Itararé-Taquara pottery are necessary in order to improve future interpretations about the movements between Southern *proto*-*Jê* groups to the coast and the *sambaqui*/*Jê* along the coast.

### A consistent fish-based diet over time

Galheta IV stands out from older coastal sites due to the absence of a shell layer [13, 39]. Shells account for less than 4% of the NISP (Number of Identified Specimens) in the zooarchaeological analysis. Still, despite the scarcity of shells, the characteristic *sambaqui* mound architecture is still observed, with numerous skeletal remains, predominantly fish, birds, and marine mammals.

Fish dominate the zooarchaeological assemblage and terrestrial fauna accounts for a small amount of the remains. The δ^13^C and δ^15^N obtained from human bones and teeth confirm the substantial reliance on marine resources by the inhabitants of Galheta IV, as previously mentioned by Colonese and colleagues [28], considering both stable isotopes and lipids analysis on pottery sherds. Marine protein appears to have played a crucial role in their diets, similar to other coastal sites of Santa Catarina [63, 118–127], a pattern also observed on the southeastern coast of Brazil [128–132].

The identified fish species suggest year-round specialized fishing techniques in the Atlantic Ocean, with the use of both fish hooks and nets. The presence of elasmobranchii specimens (sharks and rays), along with the abundance of oceanic birds and marine mammals, supports the hypothesis of strong adaptation and intense exploitation of the coastal environment, involving the use of watercrafts. Still, fishing activities might have also extended to estuarine environments, but with less frequency.

The bone isotope ratios of the individuals from Galheta IV reveal the average protein intake consumed during the last 3 to 10 years of an individual’s life. These average δ^15^N and δ^13^C values indicate a predominant consumption of marine fishes, particularly species such as the pompano fish (*Trachinotus* sp.) and lower trophic level (smaller or younger) sharks’ species. This conclusion is based on the ^15^N-enrichment (4 to 5‰, [133]) compared to these species, as well as the overlap between the values of humans and piscivores such as Odontoceti and *Shyrna* sp. Consumption of marine mammals, seabirds, and marine turtles may have also occurred but not frequently, as the expected δ^15^N values would be higher if these species were the primary dietary sources.

Compared to childhood, the diet exhibits more variability during late adolescence and early adulthood, with some individuals incorporating more C_3_ terrestrial resources (plants or animals that consume C_3_ plants). Particular emphasis on the individual of the Burial 7, which, despite having a marine diet according to the third molar dentine, exhibits a mixed diet in their bone values. A similar trend for this individual is also observed in carbon isotope ratios of the amino acids (CSIA-AA) [28].

The combination of isotopic and zooarchaeological data highlights the complexity of those populations’ relationship with the surrounding environment. Many species such as the fur seals (Otariidae) and sea birds (*Thalassarshe* sp. and *S*. *magellanicus*) are recurrent on the site but did not seem to be frequently consumed. This suggests that the faunal remains found at the site do not necessarily reflect entirely their daily diet. We hypothesize that non-fish marine animals were likely consumed in association with funerary practices.

The analysis of the zooarchaeological assemblage at Galheta IV reveals an increasing reliance on marine resources throughout the coastal occupation. This assumption is based on the larger amount of the exclusively marine fauna found at this site. Other coastal sites of the region also rely on aquatic resources, but with species that could be found both at the estuary and the sea [101, 118–120, 123–127]. However, when comparing the carbon and nitrogen isotopic ratios of humans from the non-ceramic sites Jabuticabeira II (~2400 years cal BP) [28, 124, 125, 134] and Cabeçuda (~4000 to 2800 years cal BP) [63, 124, 135], there is significant dietary overlap among most individuals. Faunal deposition differs between Galheta IV and Jabuticabeira II, with the latter showing a higher concentration of fish near human burials [125], while Galheta IV has a different distribution pattern (see next discussion session).

Additionally, the δ^15^N results from Galheta IV, Jabuticabeira II and Cabeçuda individuals presents an average ^15^N-enrichment of around 5‰ when compared with the non-ceramic *sambaquis* Caieira, Carniça I and Congonhas I [100], located in the same region and from the same time period as Jabuticabeira II and Cabeçuda (~4000 to 2200 years cal BP, [38, 102, 136]). It seems that the chronology, the absence/presence of shells and pottery are not correlated with the diets of those coastal populations. Further investigation for other sites and regions should be conducted in order to understand the origin of these differences.

It must be noted that other components of the diet, including plant species and other animals that were potentially exploited, remain unknown for Galheta IV. Feeding experiments showed that a proportion as low as 18–20% of meat in a mammal diet can entirely overprint the consumption of plants tracked by δ^13^C and δ^15^N isotopic ratios obtained from bulk collagen [137, 138]. Thus, the amount of plants consumed in Brazilian coastal archaeological contexts is therefore likely frequently underestimated [139–141]. However, several studies already showed the importance of plants and their consumption by *sambaqui* populations [134, 142–147], although isotopic techniques still struggle to detect this. To gain a more comprehensive understanding of their diets, there is a need for development of new isotope proxies less sensitive to the protein intake.

### Spatial organization and funerary practices

We found differences in the zooarchaeological composition within the site, specifically between units with and without burials. Most of the fauna is concentrated in the non-funerary units in sites located in southeastern areas, suggesting that animals, excluding cetaceans, were likely brought to the site as whole carcasses and then prepared for consumption or as part of funerary practices.

Areas near the burials yielded interesting results in terms of specific faunal remains, which appear to have been intentionally deposited close to the burials. For example, we found twelve seabird specimens (likely albatrosses) near the Burial 7. Additionally, there was a high density of burned faunal remains near the Burial 3, and unique species such as white shark (*Carcharodon carcharias*), whale (Mysticeti) and capybara (*Hydrochoerus hydrochaeris*), next to the Burials 1 and 6.

Mollusks, particularly marine gastropods (*Olivancillaria v*. *auricularia*), were mostly found near the human burials, suggesting possible symbolic purposes. Many of these gastropods exhibited a break pattern on their aperture and outer lip, indicating possible consumption.

Analysis using FTIR-ATR revealed that in Galheta IV bone color can be used as an estimate of heating temperatures. Bones that appeared unaltered were exposed to low temperatures, while human bones exhibited a range of heating temperatures, from low to moderate, suggesting varying degrees of proximity to flames but not direct and prolonged burning. In Galheta IV, we did not observe skeletal transformations typically associated with prolonged exposure to fire in cremations, such as extreme bone fragmentation, reduction, or destruction of dental crowns with enamel loss [148–150]. Since the site lacks a significant number of shells in its matrix, there were no layers acting as a barrier between the heat source and the deposited materials. Therefore, fauna, lithics, pottery, and burials were deposited at the site and subsequently experienced unintentional thermal effects.

The lack of barrier between the heating sources and the humans’ remains becomes evident in the correspondence among burned areas and bone surfaces with less muscle coverage, as well as the concentration of burned areas in bone regions closer to the surface. These findings suggest that a heat source was positioned just above the skeleton and did not involve the entire body. Considering that cremation is typically associated with intentional bodily reduction and symbolic practices, as seen in Southern *proto*-*Jê* sites [32, 33], what we observe at Galheta IV cannot be considered as such.

Most individuals found at the site were young at the time of death, although precise age estimation was challenging due to the poor preservation of many skeletal remains. In general, the funerary characteristics observed and their arrangement on the site did not correlate with variations in chronology or isotopic ratios. However, the individual of the Burial 3, one of the oldest burials at the site and representing a multiple deposition of at least four individuals, showed the highest degree of thermal alteration.

The funerary practices of the well-preserved burials at Galheta IV exhibit a deposit of the body and funerary accompaniments that are similar to what has been seen in other *sambaquis* [106, 151–154]. However, there are some differences that stand out: such as the more intense use of fire as a marker for the bodies compared to what is seen in other shellmounds; and the way in which fauna is deposited in this funerary context, accumulated away from the human remains, and the selection of specific animal species near the burials. Thus, we can identify a continuity in funerary practices of coastal sites over time, but also variabilities that are highlighted at Galheta IV.

## Conclusions

Our research represents the first multi-proxy approach that challenges the hypothesis of Southern *proto-Jê* group migration to the coast. In essence, we demonstrate that the presence of Itararé-Taquara pottery at Galheta IV does not necessarily indicate a migration of people from the hinterland. Instead, our findings suggest a scenario of continuous and prolonged contact between these populations, accompanied by localized movements of people along the coastline. This approach helps refine our understanding of the nature of interactions between *sambaqui* and Southern *proto*-*Jê* populations, offering not only new insights into the social dynamics of this period (after 1300 years ca BP) but also providing a fresh perspective on the late coastal occupation context in southern Brazil.

By investigating the diet at Galheta IV, it becomes clear that the adaptation to the ocean and the fishing activities intensifies over time. We affirm that by the abundant presence of marine species remains that are not commonly found in other shellmounds of the region. Moreover, when comparing stable isotopes data from other coastal sites, it seems that dietary variation on the southern coast of Santa Catarina was not driven by the presence of pottery, chronology or site formation changes.

The funerary practices at Galheta IV are noticeable by the use of fire as an important marker of burials depositions. However, we were able to discard the possibility of cremations. Bone and teeth tools were found mainly close to the human remains, and also specific fauna species that are not often found in other units of the site. In contrast to the findings of at Jabuticabeira II, where fish accumulations suggested feasting events near burials, Galheta IV exhibited a distinct pattern. Zooarchaeological accumulations, formed by fishes and other sea animals, were concentrated in specific areas, on the opposite side from where the burials were located.

Our study sheds light into the late occupation (after 1300 years cal BP) of the southern Brazilian coast, when the architecture of shell accumulations was abandoned after millennia. This period possibly witnessed the progressive construction of a new identity, characterized by materiality exchanges and the maintenance of old practices. The study of Galheta IV significantly contributes to the body of archaeological data, revealing that the late pre-colonial settlement of the southern coast of Santa Catarina involved a combination of resilience and change, driven by the intensification of contacts between coastal societies and Southern *proto*-*Jê* populations.

In this direction, we highlight the complexities inherent in the late period of coastal occupations. We strongly advocate for the widespread adoption of multi-proxy approaches in the exploration of diverse archaeological sites and regions. This multidisciplinary methodology, when implemented on a specific site, not only enriches our understanding by unveiling intricate details but also significantly contributes to a more comprehensive interpretation, extending its impact beyond the immediate region to broader scales of archaeological understanding.

## Supporting information

S1 Fig Archaeological sites of with late occupation at Santa Catarina coast. The highlighted sites are the ones mentioned at the main text. Made with free vector and raster map data. Background: Natural Earth; Digital elevation model: FABDEM V1-2; Rivers: Brazilian National Water Agency (ANA). All utilized geographical data are under the Creative Commons Attribution License (CC BY 4.0). Software: QGIS 3.28. Note: Own elaboration.
(TIF)

S2 Fig Calibrated radiocarbon dates for the Santa Catarina sites with Itararé-Taquara pottery. (TIF)

S1 Table Results of NISP and MNI of the taxa identified at Galheta IV archaeological site. *taxon probably non-archaeological.
(DOCX)

S1 File Spatial distribution analysis of the fauna on Galheta IV. (DOCX)

S2 File Detailed burials information. (DOCX)

S1 Dataset Dataset spreadsheets. a. Zooarchaeological analysis, b. ™^13^C and ™^15^N isotopes, c. ^87^Sr/^86^Sr isotopes, d. Trace elements. e. Pottery analysis. f. Southern Santa Catarina site’s location list, g. ^14^C of coastal Itararé-Taquara pottery sites.
(XLSX)
