# Supplementary figures and images for: Late shellmound occupation in southern Brazil: A multi-proxy study of the Galheta IV archaeological site
Source: PLoS One. 2024 Mar 21;19(3):e0300684. doi: 10.1371/journal.pone.0300684 (PMC10956814; doi:10.1371/journal.pone.0300684)

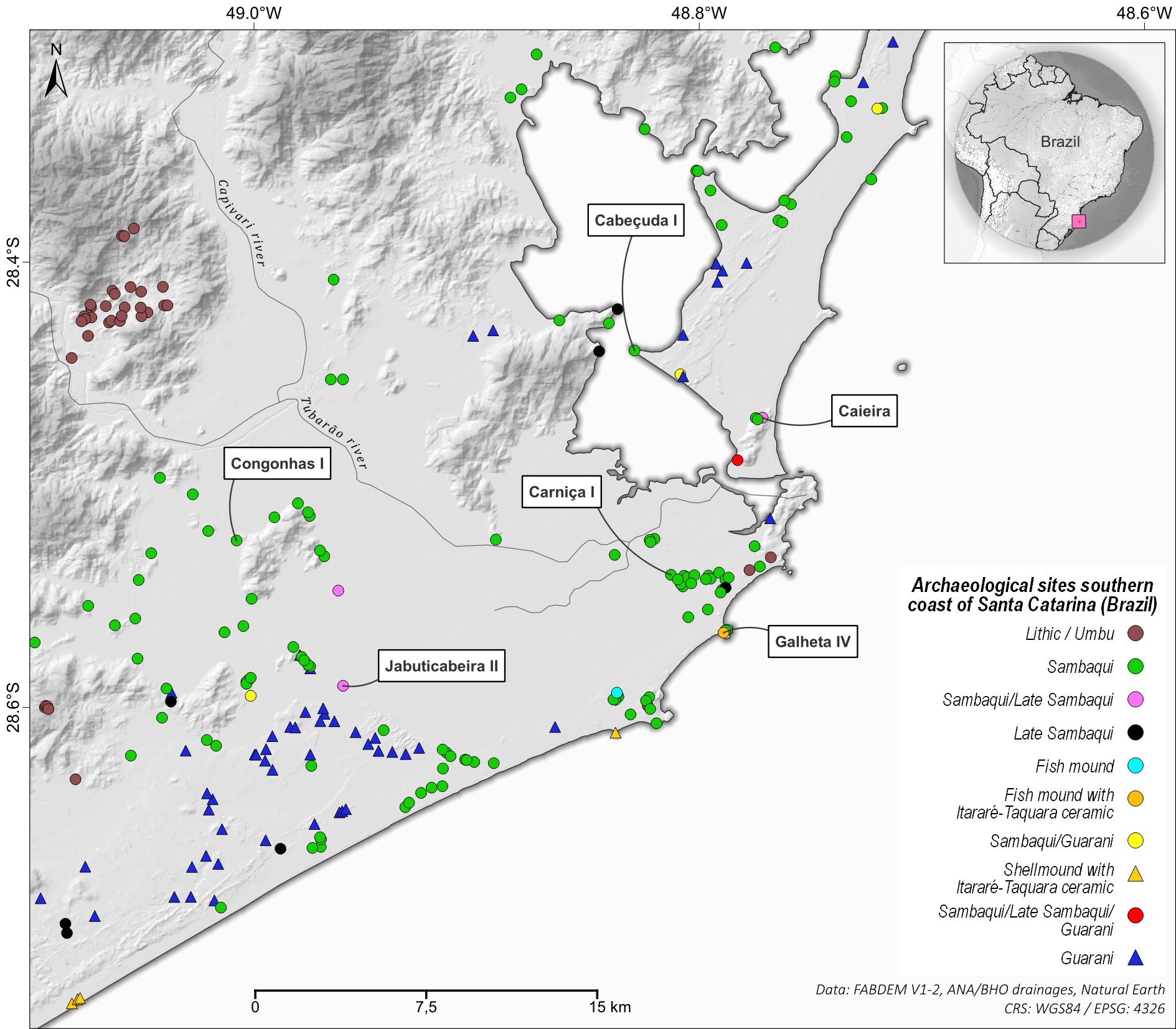

Supplement: S1 Fig — The highlighted sites are the ones mentioned at the main text. Made with free vector and raster map data. Background: Natural Earth; Digital elevation model: FABDEM V1-2; Rivers: Brazilian National Water Agency (ANA). All utilized geographical data are under the Creative Commons Attribution License (CC BY 4.0). Software: QGIS 3.28. Note: Own elaboration. (TIF) [file pone.0300684.s001.tif]

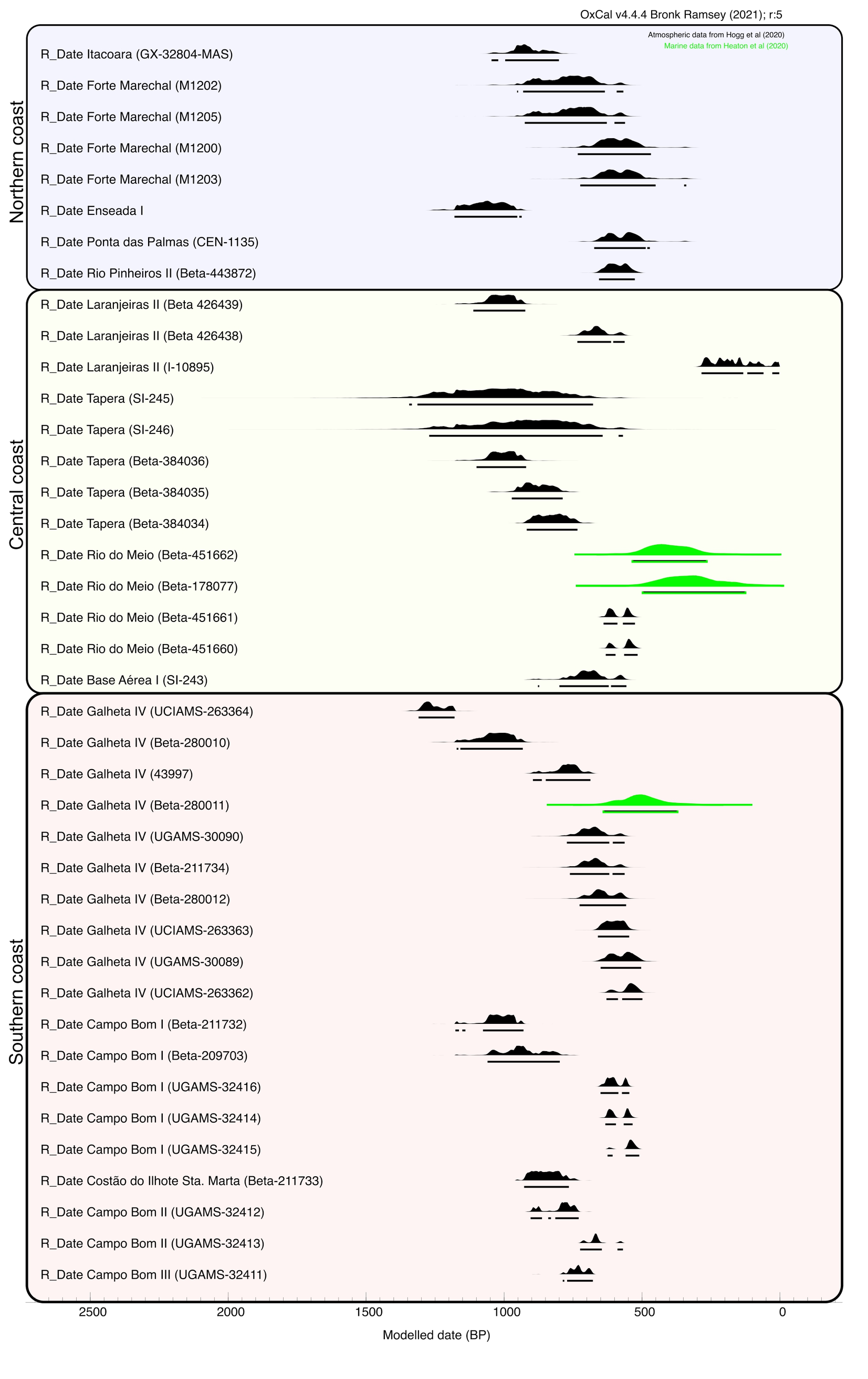

Supplement: S2 Fig — (TIF) [file pone.0300684.s002.tif]
