# Supplementary material for: Late shellmound occupation in southern Brazil: A multi-proxy study of the Galheta IV archaeological site
Source: PLoS One. 2024 Mar 21;19(3):e0300684. doi: 10.1371/journal.pone.0300684 (PMC10956814; doi:10.1371/journal.pone.0300684)
Supplement: S1 Table — *taxon probably non-archaeological. (DOCX) [file pone.0300684.s003.docx]

**Table S1. Results of NISP and MNI of the *taxa* identified at Galheta IV archaeological site.**

| ***Taxa*** | **NISP** | **%** | **MNI** | **%** |
| --- | --- | --- | --- | --- |
| **Crustacea** | 3 | 0,01 | 0 | 0,00 |
| Decapoda |  |  |  |  |
| Portunidae |  |  |  |  |
| *Callinectes* sp. | 24 | 0,10 | 6 | 0,67 |
| Hexanauplia |  |  |  |  |
| Cirripedia* | 3 | 0,01 | 3 | 0,33 |
| **Mollusca** | 279 | 1,18 | 0 | 0,00 |
| Bivalvia | 243 | 1,03 | 7 | 0,78 |
| Veneridae | 29 | 0,12 | 10 | 1,11 |
| *Amiantis purpurata* | 2 | 0,01 | 1 | 0,11 |
| *Anomalocardia flexuosa* | 4 | 0,02 | 4 | 0,45 |
| Lucinidae |  |  |  |  |
| *Phacoides pectinatus* | 5 | 0,02 | 3 | 0,33 |
| Ostreidae |  |  |  |  |
| *Ostrea* sp. | 5 | 0,02 | 2 | 0,22 |
| Gastropoda | 145 | 0,62 | 107 | 11,93 |
| Volutidae |  |  |  |  |
| *Adelomelon brasiliana* | 4 | 0,02 | 4 | 0,45 |
| Muricidae |  |  |  |  |
| *Stramonita haemastoma* | 3 | 0,01 | 3 | 0,33 |
| Olividae |  |  |  |  |
| *Olivancillaria* sp. | 2 | 0,01 | 0 | 0,00 |
| *Olivancillaria auricularia* | 81 | 0,34 | 81 | 9,03 |
| *Olivancillaria urceus* | 1 | 0,00 | 1 | 0,11 |
| **Elasmobranchii** | 236 | 1,00 | 1 | 0,11 |
| Batoidea | 67 | 0,28 | 0 | 0,00 |
| Rajiidae | 2 | 0,01 | 2 | 0,22 |
| Dasyatidae | 5 | 0,02 | 4 | 0,45 |
| Myliobatidae |  |  |  |  |
| *Rhinoptera* sp. | 1 | 0,00 | 1 | 0,11 |
| Selachii | 24 | 0,10 | 0 | 0,00 |
| Lamnidae |  |  |  |  |
| *Carcharodon carcharias* | 1 | 0,00 | 1 | 0,11 |
| Odontaspididae |  |  |  |  |
| *Charcharias taurus* | 13 | 0,06 | 4 | 0,45 |
| Carcharhinidae | 176 | 0,75 | 3 | 0,33 |
| *Prionace* sp. | 3 | 0,01 | 2 | 0,22 |
| Sphyrnidae | 56 | 0,24 | 1 | 0,11 |
| *Sphyrna* sp. | 8 | 0,03 | 4 | 0,45 |
| **Teleostei** | 7942 | 33,70 | 0 | 0,00 |
| Siluriformes |  |  |  |  |
| Ariidae | 363 | 1,54 | 23 | 2,56 |
| *Genidens barbus* | 52 | 0,22 | 14 | 1,56 |
| Mugiliformes |  |  |  |  |
| Mugilidae |  |  |  |  |
| *Mugil* sp. | 79 | 0,34 | 28 | 3,12 |
| Perciformes |  |  |  |  |
| Centropomidae |  |  |  |  |
| *Centropomus* sp. | 148 | 0,63 | 32 | 3,57 |
| Serranidae | 1 | 0,00 | 1 | 0,11 |
| Pomatomidae |  |  |  |  |
| *Pomatomus saltatrix* | 12 | 0,05 | 7 | 0,78 |
| ***Taxa*** | **NISP** | **%** | **MNI** | **%** |
| Carangidae |  |  |  |  |
| *Trachinotus* sp. | 1345 | 5,71 | 321 | 35,79 |
| Sparidae | 23 | 0,10 | 9 | 1,00 |
| Sciaenidae | 4 | 0,02 | 0 | 0,00 |
| Sciaenidae sp. Indeterminada 1 | 3 | 0,01 | 2 | 0,22 |
| *Cynoscion* sp. | 10 | 0,04 | 8 | 0,89 |
| *Micropogonias furnieri* | 681 | 2,89 | 77 | 8,58 |
| *Pogonias cromis* | 103 | 0,44 | 6 | 0,67 |
| Ephippidae |  |  |  |  |
| *Chaetodipterus faber* | 3 | 0,01 | 3 | 0,33 |
| Characiformes |  |  |  |  |
| Erythrinidae |  |  |  |  |
| *Hoplias malabaricus* | 1 | 0,00 | 1 | 0,11 |
| **Reptilia** |  |  |  |  |
| Testudines | 132 | 0,56 | 2 | 0,22 |
| Emydidae |  |  |  |  |
| *Trachemys* sp. | 25 | 0,11 | 2 | 0,22 |
| Cheloniidae | 24 | 0,10 | 4 | 0,45 |
| **Aves** | 5010 | 21,26 | 0 | 0,00 |
| Aves sp. Indeterminada 1 | 15 | 0,06 | 4 | 0,45 |
| Sphenisciformes |  |  |  |  |
| Sphescinidae |  |  |  |  |
| *Spheniscus magellanicus* | 142 | 0,60 | 13 | 1,45 |
| Procellariformes | 7 | 0,03 | 0 | 0,00 |
| Diomedeidae |  |  |  |  |
| *Thalassarche* sp. | 1020 | 4,33 | 48 | 5,35 |
| **Mammalia** | 1688 | 7,16 | 0 | 0,00 |
| Cingulata |  |  |  |  |
| Dasypodidae |  |  |  |  |
| *Dasypus novemcinctus* | 1 | 0,00 | 1 | 0,11 |
| Rodentia | 20 | 0,08 | 2 | 0,22 |
| Caviidae |  |  |  |  |
| *Hydrochoerus hydrochaeris* | 1 | 0,00 | 1 | 0,11 |
| Ctenomydae |  |  |  |  |
| *Ctenomys* *minutus** | 9 | 0,04 | 4 | 0,45 |
| Echimyidae |  |  |  |  |
| *Myocastor coypus* | 1 | 0,00 | 1 | 0,11 |
| Carnivora |  |  |  |  |
| Felidae | 1 | 0,00 | 1 | 0,11 |
| Otariidae | 257 | 1,09 | 0 | 0,00 |
| *Arctocephalus* sp. | 250 | 1,06 | 8 | 0,89 |
| *Arctocephalus australis* | 40 | 0,17 | 7 | 0,78 |
| Artiodactyla |  |  |  |  |
| Tayassuidae | 3 | 0,01 | 3 | 0,33 |
| Cervidae |  |  |  |  |
| *Mazama* sp. | 1 | 0,00 | 1 | 0,11 |
| *Ozotocerus bezoarticus* | 1 | 0,00 | 1 | 0,11 |
| Cetacea | 218 | 0,93 | 5 | 0,56 |
| Mysticeti | 4 | 0,02 | 1 | 0,11 |
| **Unknown** | 2498 | 10,60 | 0 | 0,00 |
| **TOTAL** | 23564 | 100 | 897 | 100,00 |

**taxon* probably non-archaeological
