# Supplementary material for: Late shellmound occupation in southern Brazil: A multi-proxy study of the Galheta IV archaeological site
Source: PLoS One. 2024 Mar 21;19(3):e0300684. doi: 10.1371/journal.pone.0300684 (PMC10956814; doi:10.1371/journal.pone.0300684)
Supplement: S1 File — (DOCX) [file pone.0300684.s004.docx]

# Spatial distribution of the fauna on Galheta IV

The NISP (Number of Identified Specimens) and MNI (Minimum Number of Individuals) data were grouped into taxonomic families and then subjected to X² statistical tests between the different analyzed contexts, aiming to measure the chances of taxon distribution being random and establish comparative interpretations within intra-site areas. The results of the tests based on these two variables (NISP and MNI) showed differences because the NISP estimates of taxa presented higher values, as it considers identified pieces, while the MNI estimates presented lower values, as it represents the minimum number of individuals present.

The results obtained from the NISP are shown in following table and presented statistical differences among all the studied areas/quadrants (p<0.05). Specimens from the Carangidae family (jacks) indicate the most significant differences among the analyzed areas, followed by Diomedeidae (albatrosses). Otariidae (seals) also play a significant role in highlighting the statistical differences between quadrant 112/93 (area with concretions) and quadrant 111/99, area B, and Profile (areas associated with burials). Meanwhile, differences observed between quadrant 110/94 (without defined association) and the others are mainly defined by the identified pieces of Sciaenidae (croakers), Ariidae (catfish), and Cetacea (whales and dolphins).

Table S2. Results of the comparative test between the areas/quadrants based on NISP data.

|  | **Area A 110-94** | **Area A 111-99** | **Area A 112-93** | **Area B** | **Profile** |
| --- | --- | --- | --- | --- | --- |
| **Area A 110-94** |  | X²= 198,87  ddl= 11 p= 0,0001 Diomedeidae, Carangidae e Cetacea | X²= 192,59  ddl= 11 p= 0,0001  Carangidae, Diomedeidae e Sciaenidae | X²= 124,68  ddl= 11 p= 0,0001 Carangidae, Diomedeidae e Sciaenidae | X²= 70,219  ddl= 11 p= 0,0001 Carangidae, Ariidae e Diomedeidae |
| **Area A 111-99** |  |  | X²= 647,86  ddl= 14 p= 0,0001 Carangidae, Diomedeidae e Otariidae | X²= 197,62  ddl= 13 p= 0,0001 Cetacea, Carangidae e Diomedeidae | X²= 94,922  ddl= 12 p= 0,0001 Diomedeidae, Carangidae e Sciaenidae |
| **Area A 112-93** |  |  |  | X²= 353,23 ddl= 17 p= 0,0001 Carangidae e Otariidae | X²= 266,53  ddl= 14 p= 0,0001 Carangidae, Otariidae e Diomedeidae |
| **Area B** |  |  |  |  | X²= 77,753  ddl= 13 p= 0,0001 Carangidae, Diomedeidae e Sciaenidae |
| **Profile** |  |  |  |  |  |

The results of X², degrees of freedom (ddl), p-value, and the families that showed the greatest significant differences among the analyzed contexts.

On the other hand, the results obtained from the MNI demonstrated that the probability of similarities in the distribution of materials between quadrant 110/94 (without defined association) and area B, Profile, and quadrant 111/99 (areas associated with burials) is higher. Meanwhile, statistically significant differences are shown between quadrant 112/93 (area with concretion) and Profile and quadrant 111/99 (areas associated with burials), as well as quadrant 110/94 (without defined association). However, the area with concretion shows greater similarities with area B, which contains burials. The taxonomic family that defines the statistical differences among the mentioned contexts is primarily characterized by the NMI values of the Carangidae.

Table S3. The comparative test results between the areas/quadrants based on the MNI data.

|  | **Area A 110-94** | **Area A**  **111-99** | **Area A**  **112-93** | **Area B** | **Profile** |
| --- | --- | --- | --- | --- | --- |
| **Area A 110-94** |  | X²= 0,16171 ddl= 2 p= 0,9586 | X²= 33,296 ddl= 2 p= 0,0001 Carangidae | X²= 1,3001 ddl= 2 p= 0,494 | X²= 4,2718 ddl= 2 p= 0,1322 |
| **Area A 111-99** |  |  | X²= 30,771 ddl= 4 p= 0,0001 Carangidae | X²= 16,225 ddl= 5 p= 0,0057 Carangidae | X²= 5,6791 ddl= 5 p= 0,3486 |
| **Area A 112-93** |  |  |  | X²= 10,455 ddl= 5 p= 0,0632 | X²= 39,263 ddl= 6 p= 0,0001 Carangidae |
| **Area B** |  |  |  |  | X²= 18,593 ddl= 6 p= 0,004 Carangidae |
| **Profile** |  |  |  |  |  |

The X² results, degrees of freedom (ddl), p-value, and the families that exhibited the highest significant differences among the analyzed contexts.

A hierarchical cluster analysis test (Classical Clustering using Euclidean distance) was also conducted in PAST using the NISP data obtained for taxonomic families. The results showed that Profile (area associated with burials) and Area A 110/94 (without defined association) exhibited greater proximity, followed by quadrant 111-99 and Area B (areas associated with burials). The concretion areas, represented by quadrants 112-93 and 113-95, were further apart from the others. The *taxa* that defined these distances were: Spheniscidae, Bivalves, Centropomidae, Testudines, Gastropoda, Batoidea, Sciaenidae, Diomedeidae, Ariidae, Charcarhinidae, and Carangidae.


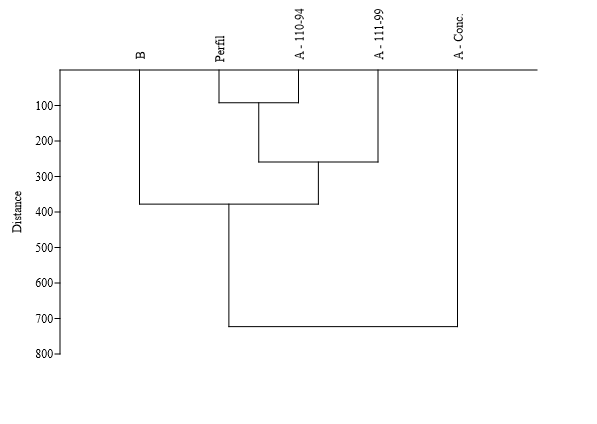


**Figure S4. The result of the hierarchical analysis (Clustering) based on NISP data of the areas** (n = 4631).
