# Supplementary material for: Late shellmound occupation in southern Brazil: A multi-proxy study of the Galheta IV archaeological site
Source: PLoS One. 2024 Mar 21;19(3):e0300684. doi: 10.1371/journal.pone.0300684 (PMC10956814; doi:10.1371/journal.pone.0300684)
Supplement: S2 File — (DOCX) [file pone.0300684.s005.docx]

# Detailed burials information

## Burial 1

**Figure S5.** **Burial 1 during excavation.**

Location: Area B of the excavation, at level 1 (layer 02) unit 102/107.

Radiocarbon dating: 744-563 cal BP (Beta 211734 – 980 + 40 BP) (1).

Sex: Undetermined. Unpreserved markers. The pelvis was not preserved, only a fragment of the ilium and acetabulum are present, and the skull does not have preserved markers.

Age: 17 to 19 years. The age was estimated considering the fusion of epiphyses and dentition. The individual has all erupted teeth, but the third molars do not show wear. Regarding fusion, all analyzed epiphyses were still in the process of fusion, including the clavicles, left scapula, radius, ulna, metacarpals, and phalanges of the hands, femurs, tibias, and fibulas. The markers used were those indicated by Buikstra & Ubelaker (1994) (2) and Black & Scheuer (2000) (3).

Body deposition: Primary and simple burial, deposited in a left lateral decubitus position, with articulated bones. In general, the skull appears to be resting on the left shoulder, the upper limbs have the arms aligned on the lateral side of the thoracic region, forearms and hands slightly flexed over the region between the abdomen and pelvis. The lower limbs are flexed, and the feet are not observable in the photographic record.

Context/funerary accompaniment: A series of adornments made from fauna were found near burials 1 and 6: five perforated shark vertebrae, a robust 9 cm bone tip from a mammal, and 3 fragmented tips of bird bones. Additionally, unique species such as white shark vertebrae (*Carcharodon carcharias*), a big whale vertebrae bone (Mysticeti), and a capybara incisor (*Hydrochoerus hydrochaeris*) were discovered.

Taphonomy: Longitudinal and transverse fractures to the bone's major axis. Thermal alterations caused by fire, present in some bones.

Heating estimation: During curation, it was observed that some bones may have undergone thermal alterations, resulting in differences in color, weight, and the presence of longitudinal fissures in the long bones. In burial 1, only the patellae demonstrated a change in color (dark brown), while the other bones show fissures to a greater or lesser extent. Considering the position of the individual (left lateral decubitus), it is possible to perceive that the major fissures are located in the lateral and anterior portions of the right limbs and the fragment of the right frontal bone and mandible. The estimated heating temperature by FTIR analysis was >600°C.

Additional information: Burial 1 was superimposed on Burial 6 (Area B). The dates indicate that both burials were deposited in very close periods and could have been deposited simultaneously. During curation, it was noted that Burial 1 contains fragments of the skull belonging to 03 individuals and maxillae belonging to two individuals. In the laboratory, it was not possible to determine to which other disturbed burials these bones could be associated.

**
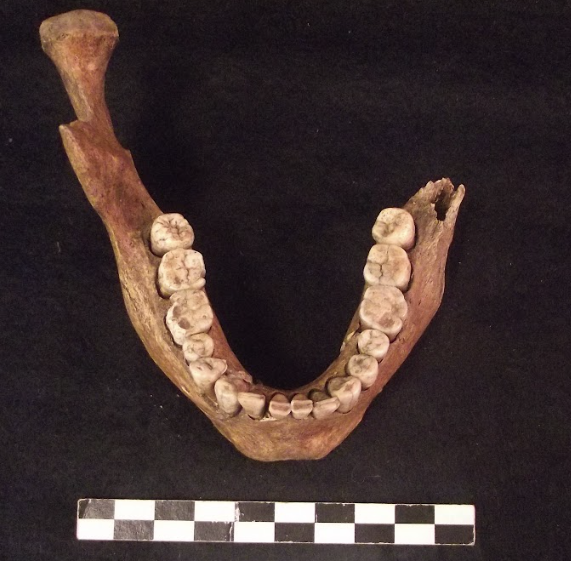

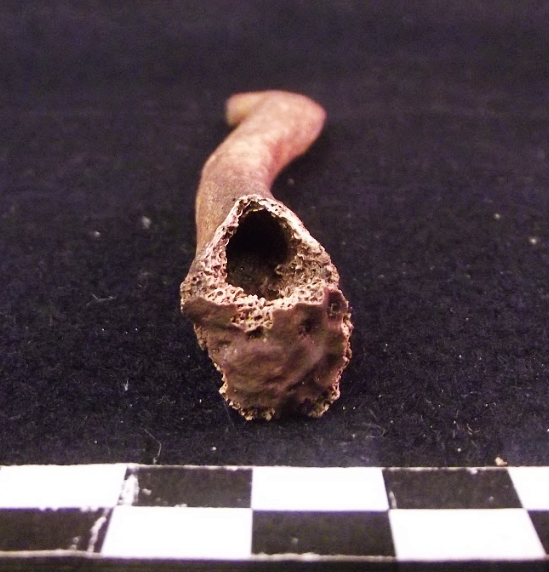
**

**Figure S6. Burial 1. Mandible and right clavicle with sternal extremity non-fused.**

## Burial 2

Location: Area A of the excavation, at level 1 (layer 2) of units 110/97 and 111/97.

Radiocarbon dating: 629-498 cal BP (UCIAMS 263362 – 805 + 15 BP).

Sex: Undetermined. Absence of marker bones for estimation, due to the limited number of bones present in the burial.

Age: Undetermined.

Body deposition: The individual's bones were disarticulated and disorganized, according to the written field record. It only contains some long bones, such as the left femur and fibula, in addition to some fragments of the skull. Based on the field records and curation, it may be a secondary and simple burial; however, the photographic record is not available, which hinders clear identification and inferences.

Context/funerary accompaniment: no record.

Taphonomy: Thermal alterations caused by fire; transverse fractures to the bone's major axis. Absence of adequate elements for the estimation of macroscopic burning, due to the limited number of bones present in the burial.

Heating estimation: The bones show marks caused by the heat of the fire, such as changes in coloration and bone expansion indicating they underwent a burning process, but they did not experience direct exposure to fire. The estimated heating temperature by FTIR analysis was >600°C.

Additional information: In the spatial distribution of Area A, Burial 2 (unit 111/97) was close to Burial 9 (unit 110/108) and Burial 7 (unit 111/99).


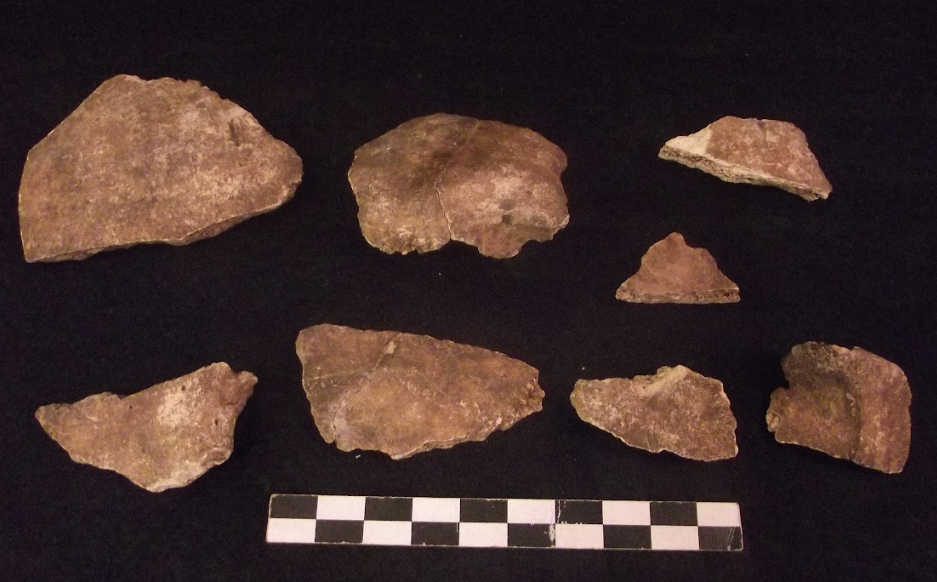


**Figure S7. Burial 2. Fragments of cranium bones.**

## Burial 3

**Figure S8. Burial 3 during excavation.**

Location: Area B of the excavation, at level 1 (layer 2) of unit 107/109.

Radiocarbon dating: 1173-931 cal BP (Beta 280010 – 1360 + 40 BP) (1).

Sex: Undetermined though bioarchaeological marks, the lack of skull and pelvic markers makes it impossible to estimate the sex for the two individuals (Individual 3A and 3B). Determined as female by DNA studies (4).

Age:

Individual 3A: Undetermined. Due to the presence of few bones, the age of this individual was only estimated for the age class (adult), considering the size and robustness of the bone fragments of the skull and femur.

Individual 3B: 14 to 16 years old. The presence of erupted 1st and 2nd upper premolars, as well as epiphyseal fusion in metacarpals, ulna, and femurs, were the markers used to estimate the age. The markers used were those indicated by Buikstra & Ubelaker (1994) (2) and Black & Scheuer (2000) (3).

Body deposition: Secondary and multiple burial of at least 2 individuals, with the possibility of being composed of 4 individuals*. It is not possible to infer the arrangement and organization of this set of bones in detail, although photographic records show that some bones could be partially articulated. Taphonomy: Thermal alterations caused by fire; transverse and longitudinal fractures to the bone's major axis.

Context/funerary accompaniment: a several faunal bones were found burned close to this burial. Two bone tips made of bird bone was found in this context.

Heating estimation: The bones in this burial have a darker color compared to the bones of other burials at the site. The estimated heating temperature by FTIR analysis was >800°C (individual 3B).

Additional information: *A disjointed set of bones was identified next to Burial 3 (Individuals 3A and 3B). The size and robustness of these bones do not match any of the identified individuals; this disjointed set is composed of three fragments of tibia. Additionally, bones of ulna, vertebra, and pelvis related to a enfant were found in the fauna samples.

**
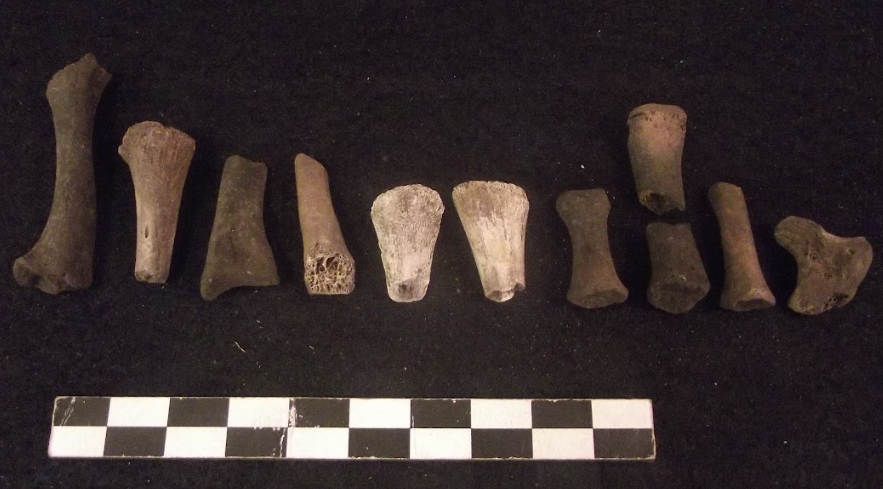
** **
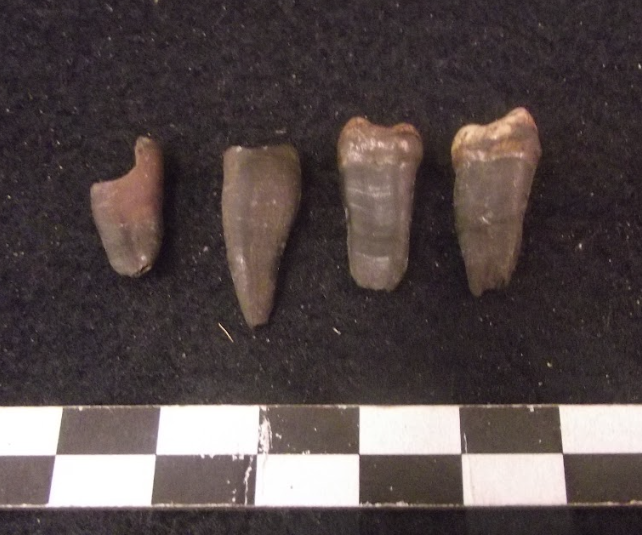
**

**Figure S9. Burial 3. Hand bones and teeth. Important to observe the different coloration dur to heating exposure, even on teeth roots.**

## Burial 4


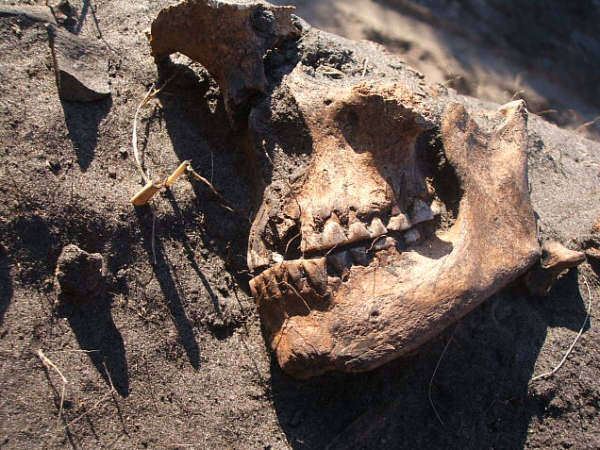


**Figure S10. Burial 4 during excavation.**

Location: Area A of the excavation, at level 1 (layer 2) of units 107/98 and 107/97.

Radiocarbon dating: 650-502 cal BP (UGAMS 30089 - 830 + 43 BP).

Sex: Undetermined. The lack of skull and pelvic markers makes it impossible to estimate.

Age: ± 20 years. The individual has all upper teeth erupted, including the third molars. Regarding epiphyseal fusion, the acromion of the right scapula, the proximal epiphysis of the radius, and the distal epiphyses of the ulna and humerus were observed, all of which are fused. In the vertebrae, it is possible to observe the vertebral ring still in fusion and growth finalization, indicating a young individual. The markers used were those indicated by Buikstra & Ubelaker (1994) (2) and Black & Scheuer (2000) (3).

Body deposition: Simple primary burial, in a right lateral decubitus position, with the right side of the body at lower levels compared to the left side, mainly in the upper portion of the body. Most of the skull was cut by the road profile and is not present. The left arm is extended alongside the body with the forearm slightly flexed and supported in the abdomen/pelvic region, and the hand cannot be observed in the field record (although some fragments were identified in the laboratory). The right arm is beside the body with the elbow joint flexed, and the hand is not visible. The lower limb is flexed, and the feet cannot be observed in the field photographic record (but some fragments were identified in the laboratory).

Context/funerary accompaniment: a fusiform artifact made of a deer antler was found above this burial.

Taphonomy: Thermal alterations caused by fire; longitudinal and transverse fractures to the bone's major axis.

Heating estimation: The bones of skull fragments, ribs, vertebrae, pelvis, feet, hands, and long bones show marks of action caused by heat from the fire, such as changes in coloration and bone expansion, indicating that they underwent a burning process, although not directly exposed to the fire. The estimated heating temperature by FTIR analysis was >500°C.

Additional information: The description of body deposition was based on the photographic record.

**
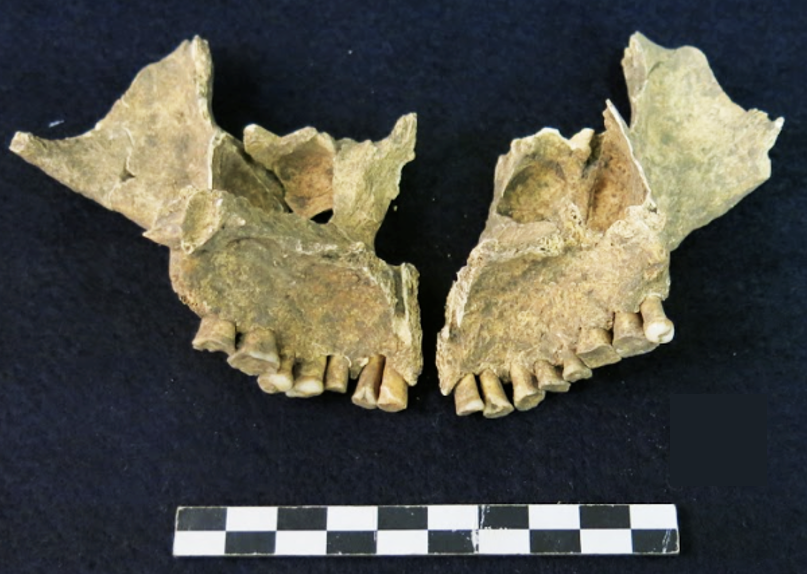
**

**Figure S11. Burial 4. Maxilla showing the left third molar erupted.**

## Burial 5

Location: Area A of the excavation, at level 1 of unit 113/93.

Radiocarbon dating: 893-685 cal BP (Curt-Engelhorn-C,entre for Archaeometry, Mannheim 43997 – 1105 + 17 BP) (4).

Sex: Undetermined by bioarchaeological markers. The sex could not be identified due to the lack of pelvic and cranial markers. Later, genetic analysis identified the sex as female (4).

Age: ± 21 years. The age was estimated considering the complete fusion of the identified metacarpal epiphyses and the presence of the erupted left upper third molar. The markers used were those indicated by Buikstra & Ubelaker (1994) (2) and Black & Scheuer (2000) (3).

Body deposition: This burial contains only some cranial bones, maxilla, mandible, teeth, fragments of vertebrae, and some hand bones, all of which belong to the same individual. However, the field record does not allow for inferences regarding the arrangement and relationship of the found bones.

Context/funerary accompaniment: no record.

Taphonomy: Thermal alterations caused by fire.

Heating estimation: The bones of the skull and mandible show evidences of heat from the fire, such as changes in coloration and bone expansion, indicating that they underwent a burning process, although not directly exposed to the fire. The estimated heating temperature by FTIR analysis was >400°C.

Additional information: Alongside the bones of burial 5, a bone (fragment of axis) was identified, which does not belong to this burial and appears to be a repetition.


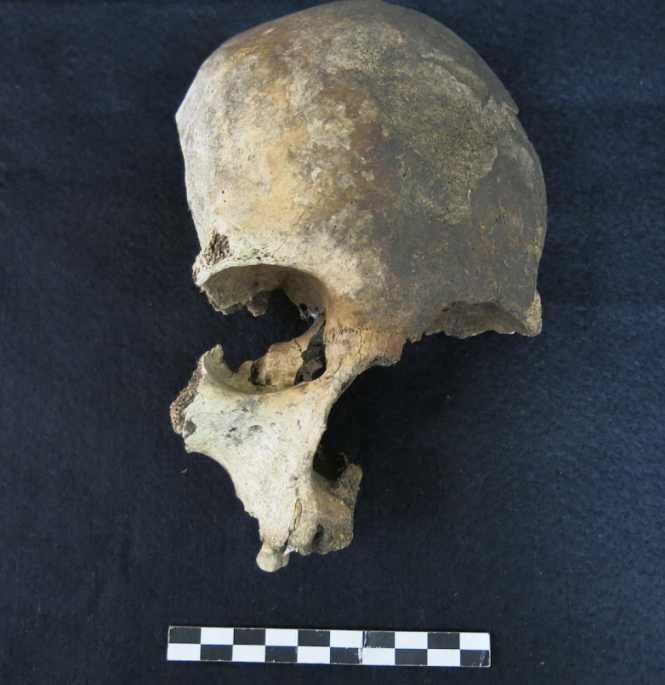


**Figure S12. Burial 5. Skull showing heating exposure evidences.**

## Burial 6

**Figure S13. Burial 6 during excavation.**

Location: Area B of the excavation, at level 2 of units 102/107, 102/108, 103/108.

Radiocarbon dating: 769-563 cal BP (UGAMS 30090 – 990 + 44 BP).

Sex: Undetermined. The sex could not be estimated due to the lack of pelvic and cranial markers.

Age: > 25 years. The analyzed epiphyses for age estimation were the acromion of the scapula, proximal and distal epiphyses of the radius, tibia, and fibula, all of which are fused. The markers used were those indicated by Buikstra & Ubelaker (1994) (2) and Black & Scheuer (2000) (3).

Body deposition: Simple primary burial. The excavation record describes that the upper part of the burial was identified and excavated first, and the lower part was excavated later, but only the tibiae, fibulae, and feet were found in a position compatible with the dorsal decubitus. The upper set of bones has the forearms and hands in the expected anatomical relationship, but the humerus is displaced and located in the central part of the chest, with the mandible between the hands. The bones in the lower part of the burial are in anatomical position and articulated, but the femurs are not present. Overall, based on the photographic record, the burial appears to be extended and in a dorsal position, but unfortunately, the relationship and arrangement between the upper and lower portions of the individual cannot be identified from the images; this relationship is only established in the written records.

Context/funerary accompaniment: A series of adornments made from fauna were found near burials 1 and 6: five perforated shark vertebrae, a robust 9 cm bone tip from a mammal, and 3 fragmented tips of bird bones. Additionally, unique species such as white shark vertebrae (*Carcharodon carcharias*), a big whale vertebrae bone (Mysticeti), and a capybara incisor (*Hydrochoerus hydrochaeris*) were discovered. Specifically close to burial 6, it was found 3 perforated shark teeth, two were *Carcharodon carcharias* and one of *Sphyrna* sp.; and also, a unilateral polished tip made of mammal bone.

Taphonomy: Thermal alterations caused by fire; longitudinal and transverse breaks to the major axis of the bone.

Heating estimation: The bones show marks caused by the heat of the fire, such as changes in coloration and bone expansion, indicating that they underwent a burning process, although not directly exposed to the fire. During the curation process, it was observed that the bones had altered coloration only in some parts, and the position of these burn marks, when considering the position of the bones observed in the photographic record, corresponds to the bone surface closest to the surface of the site (for example, in the case of the bones of the lower limbs, the alteration is observed on the anterior part of the bones). The estimated heating temperature by FTIR analysis was >400°C.

Additional information: Burial 6 was below burial 1 (Area B), and the dating indicates that the two burials were deposited in very close periods and could even have been deposited simultaneously.


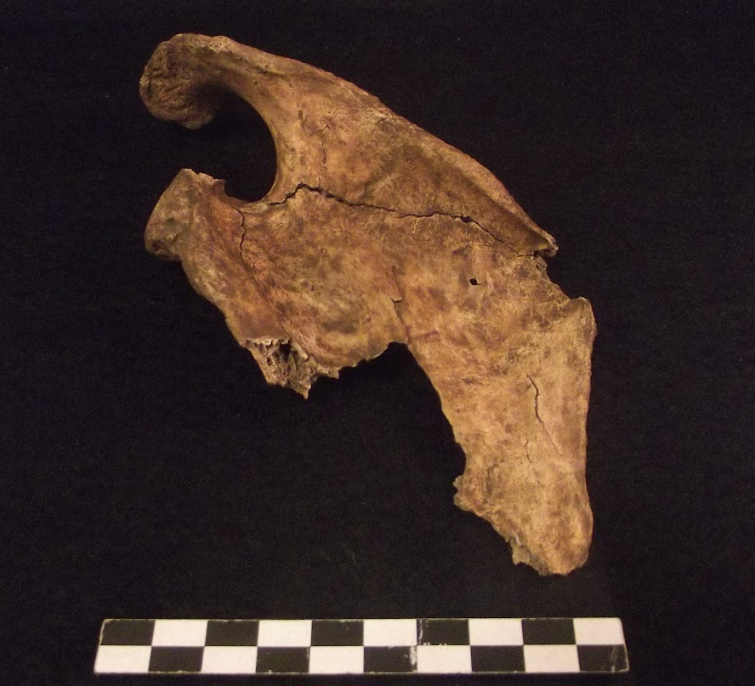

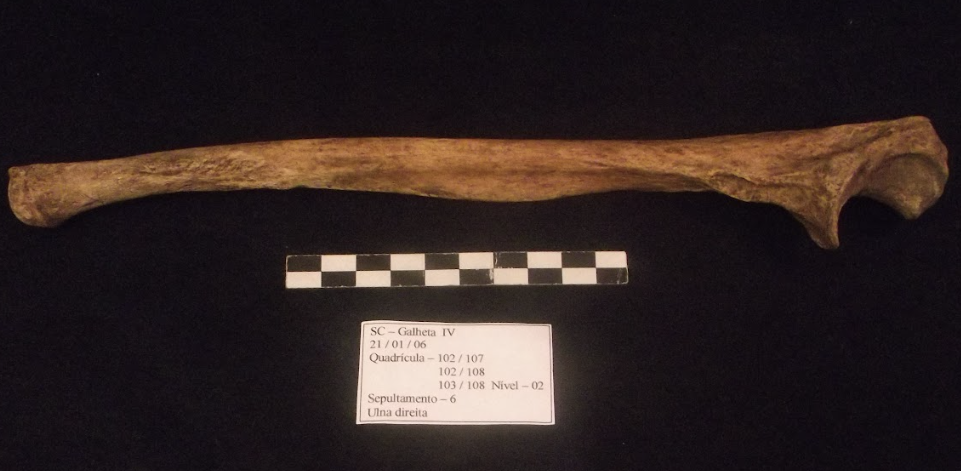


**Figure S14. Burial 6. Left scapula and left ulna.**

## Burial 7

**Figure S15. Burial 7 during excavation. The human bones were surrounded by seabird long bones.**

Location: Area A of the excavation, at level 2 of unit 111/99.

Radiocarbon dating: 725-555 cal BP (Beta 280012 – 950 + 40 BP) (1).

Sex: Undetermined. The sex could not be estimated due to the lack of pelvic and cranial markers.

Age: 19 to 20 years. The age was estimated based on the fusion of the distal epiphysis of the humerus, the distal epiphysis of the fibula, and the presence of the fusion line of the ramal epiphysis of the ischium. Regarding dentition, the individual has erupted third molars. The markers used were those indicated by Buikstra & Ubelaker (1994) (2) and Black & Scheuer (2000) (3).

Body deposition: The bones were disorganized and disarticulated, as recorded in the field, and the skeleton is incomplete and highly fragmented according to laboratory analysis. It is likely a secondary burial.

Context/funerary accompaniment: this burial was found covered by seabird bones. The zooarchaological analysis points out to at least 12 bird specimens (likely albatrosses – *Thalassarche* sp.). Besides that, three artifacts made of sharks vertebrae was found close to the human bones; and also two bone tips of bird long bones.

Taphonomy: Thermal alterations caused by fire; longitudinal and transverse breaks to the major axis of the bone.

Heating estimation: The bones of the skull, maxilla, mandible, ribs, pelvis, and fragments of long bones show marks caused by the heat of the fire, such as changes in coloration and bone expansion, indicating that they underwent a burning process, although not directly exposed to the fire. The estimated heating temperature by FTIR analysis was >400°C.

Additional information: In the laboratory, a set of bones not belonging to burial 7 was identified, consisting of a fragment of a clavicle – this fragment, in comparison with individual 7, is larger and more robust. The epiphysis of this fragment of the clavicle is fused, indicating an age range higher than estimated for individual 7.


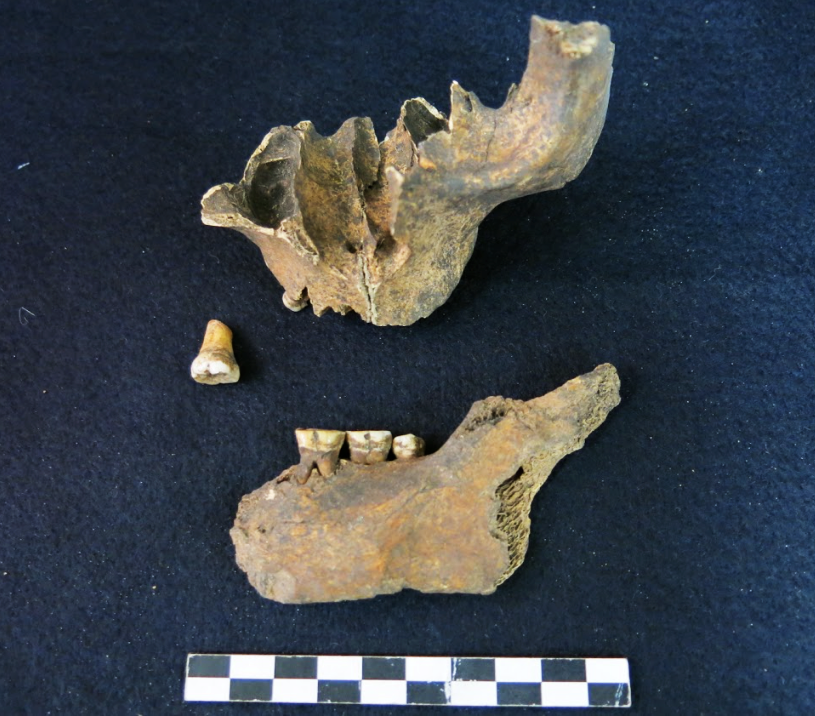

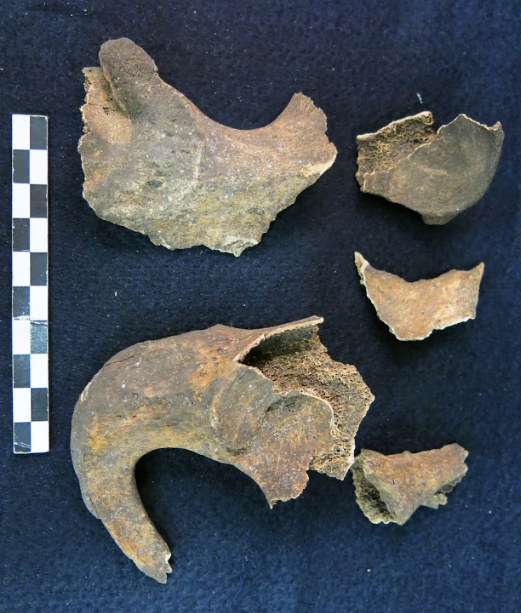
**Figure S16. Burial 7. Maxilla and mandibula with third molars erupted; fragments of pelvis.**

## Burial 8

This burial was identified in the profile, but was not excavated.

## Burial 9


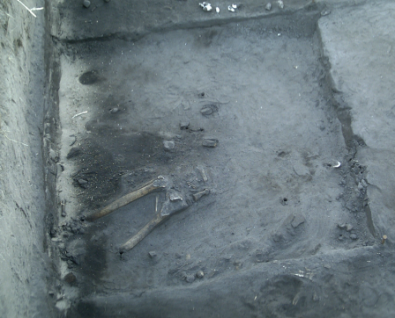


**Figure S17. Burial 9 during excavation.**

Location: Area A of the excavation, at level 2 (layer 2) of unit 110/98.

Radiocarbon dating: 658-546 cal BP (UCIAMS 263363 – 885 + 15 BP).

Sex: Undetermined. Absence of markers for estimation due to the few bones present in the burial.

Age: Undetermined. Absence of markers for estimation due to the few bones present in the burial.

Body deposition: The records of the burial do not allow identifying either the type of deposition or the body's position.

Context/funerary accompaniment: no record.

Taphonomy: Thermal alterations caused by fire; transverse breaks to the major axis of the bone.

Heating estimation: All the bones present, right femur, left tibia, and left fibula, show marks caused by the heat of the fire, such as changes in coloration and bone expansion, indicating that they underwent a burning process, although not directly exposed to the fire. The estimated heating temperature by FTIR analysis was >400°C.

Additional information: The burial contains only the long bones of the lower limbs: right femur, left fibula, and left tibia.


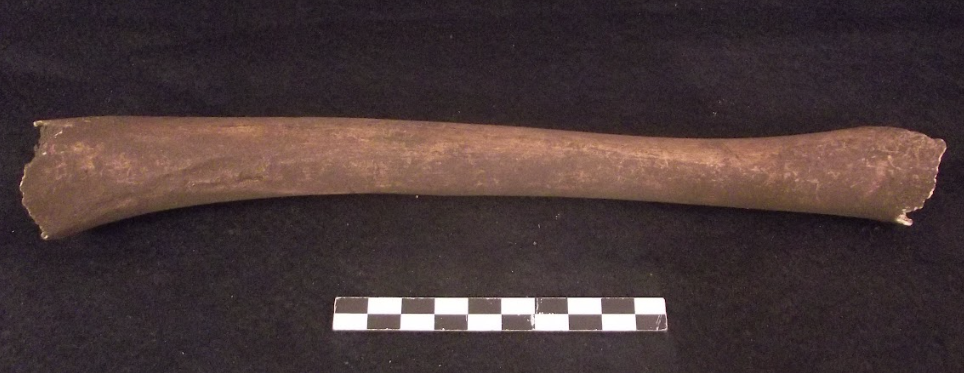


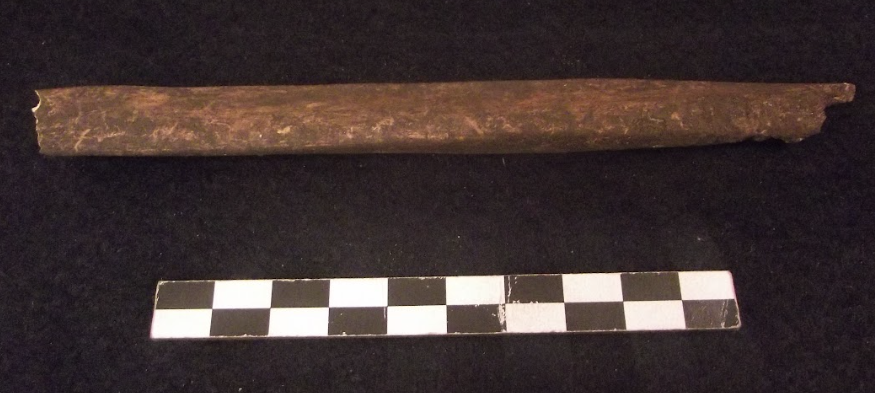


**Figure S18. Burial 9. Lower limb bones: left tibia and fibula.**
